# Clinical characteristics of autoimmune encephalitis with co-existence of multiple anti-neuronal antibodies

**DOI:** 10.1186/s12883-023-03514-x

**Published:** 2024-01-02

**Authors:** Yiyi Zhou, Hao Chen, Min Zhu, Menghua Li, Lianqun Wang, Zunchun Xie, Meihong Zhou, Xiaomu Wu, Daojun Hong

**Affiliations:** Department of Neurology, The First Affiliated Hospital, Jiangxi Medical College, Nanchang University, Nanchang, 330006 China

**Keywords:** Autoimmune encephalitis, Anti-neuronal antibody, Multiple antibodies, Autoantibodies, Disease prognosis

## Abstract

**Background:**

An increasing number of cases of autoimmune encephalitis (AE) with co-existing multiple anti-neuronal antibodies have been reported in recent years. However, the clinical significance of the concurrent presence of multiple anti-neuronal antibodies in patients with AE remains unclear.

**Methods:**

We retrospectively enrolled AE patients with multiple anti-neuronal antibodies treated at our center between August 2019 and February 2022. We also reviewed cases reported in multiple literature databases. Preferred Reporting Items for Systematic Reviews and Meta-Analyses (PRISMA) guideline was followed on selection process. And then the clinical and laboratory data of these cases were collected for review and summary.

**Results:**

A total of 83 AE cases with multiple antibodies (9 cases from our center and 74 cases from the literatures reviewed) were identified. In our center, nine patients presented with encephalitis symptoms, clinically characterized as disturbed consciousness, seizures, cognitive impairment, and psychiatric disorders. Of the 83 cases, 73 cases had co-existence of 2 types of antibodies, 8 cases had 3 types, and 2 cases had 4 types. Thirty-nine cases (39/83, 46.9%) were confirmed or suspected of also having a tumor, of which the most common was lung cancer (28/83, 33.7%). Partial or complete recovery was achieved in 57 cases (57/83, 68.6%), while 26 cases (26/83, 31.3%) died during treatment or follow-up.

**Conclusions:**

AE with co-existing multiple anti-neuronal antibodies is a specific subgroup, that is increasingly recognized in clinical practice. The co-existence of multiple anti-neuronal antibodies has a major impact on clinical features, disease progression, and prognosis.

**Supplementary Information:**

The online version contains supplementary material available at 10.1186/s12883-023-03514-x.

## Introduction

Autoimmune encephalitis (AE) usually refers to a single anti-neuronal antibody-mediated encephalopathy syndrome [1]. The clinical features of AE are characterized mainly by extensive brain parenchymal dysfunction, including disturbed consciousness, seizures, cognitive impairment, movement disorders, and psychiatric symptoms [1]. Since the first discovery of N-methyl-D-aspartate receptor (NMDAR) antibodies by Dalmau et al. in 2007 [2], anti-neuronal antibodies associated with AE have been found increasingly. At present, auto-antibodies related to AE can be divided into two categories: (1) Against the surface receptors of neurons, mainly including NMDAR, gamma aminobutyric acid receptor (GABA_B_R), leucine-rich glioma-inactivated 1 (LGI1), alpha-amino-3-hydroxy-5-methyl-4-isoxazolepropionic acid receptor (AMPAR), contactin- associated protein-2 (CASPR2), dipeptidyl-peptidase-like protein 6(DPPX), IgLON5, glycine receptor (GlyR), glutamate receptor 5 (GluR5), and dopamine-2 receptor (D2R); (2) Against the intracellular antigens of neurons, mainly including Hu, Yo, Ri, Tr, SRY-related HMG-box gene 1 (SOX1), Ma1, Ma2, glutamate decarboxylase (GAD), and CV2, which are also referred to as paraneoplastic syndrome related antibodies [3].

An anti-neuronal antibody usually corresponds to a distinct neurological syndrome, and has great specificity and influence on the diagnosis and treatment of AE [4]. An increasing number of AE cases with co-existence of multiple anti-neuronal antibodies have been reported following the growing use and advances in recent years of techniques used to detect antibodies. However,the impact of the presence of multiple antibodies on the clinical manifestations, imaging alterations, and prognosis of AE remains unclear. The presence of these multiple antibodies poses great challenges for clinical practice. The objective of the current study was therefore to investigate the clinical significance of the concurrent presence of multiple anti-neuronal antibodies by analyzing the clinical characteristics and laboratory data of patients with AE with multiple anti-neuronal antibodies treated at our center and those of patients obtained from a search of published literature.

## Materials and methods

### Subjects

We retrospectively collected AE patients with co-existence of multiple anti-neuronal antibodies who were treated from August 2019 to February 2022 in the Department of Neurology, the first affiliated hospital of Nanchang University. Every participant provided informed consent to participate in the study under the terms of the ethical approval. Patients with AE coexisting with the glial fibrillary acidic protein (GFAP) antibody or auto-antibodies associated with demyelination of the center nervous system (CNS), including myelin oligodendrocyte glycoprotein (MOG), aquaporin-4 (AQP4), and myelin basic protein (MBP) were excluded. Initial symptoms, age of onset, clinical manifestations, radiological changes, treatments, and prognosis were collected from their relatives and medical records.

### Patient consent statement

The study was approved by the Ethics Committee of the first affiliated hospital of Nanchang University. All the samples were obtained after written, signed consent was obtained from each family in compliance with the bioethical laws of China as well as the Declaration of Helsinki.

### Brain MRI

All magnetic resonance image (MRI) examinations were performed as routine clinical care using a 3.0T MR scanner with a 32-channel head/neck coil (Signa Pioneer, GE, Healthcare, USA). The images were interpreted by radiologists specialized in medical imaging. Conventional T1-weighted spin echo (T1WI) sequences, T2-weighted spin echo (T2WI) sequences, fluid attenuated inversion recovery (FLAIR) sequences, and diffused weight image (DWI) sequences were conducted according to routine procedures.

### Measurement of antibodies

Serum and cerebrospinal fluid (CSF) samples were collected from all the patients. The antibody panels of anti-neuronal surface antigens and intracellular antigens were supported commercially by Kindstar Global Inc. (Wuhan, China). Positive control and negative controls were set-up for each test. If a positive antibody were detected in a patient, it was re-checked. No tissue-based assay (TBA) was performed in the positive patients.

In brief, antibodies against neuronal cell-surface antigens were detected in vitro by the cell transfection assay using the IFT Kit MT285-16 (Shanxi MYBiotech Co., Ltd. China). This included detection of IgG auto-antibodies to NMDAR, AMPAR1, AMPAR2, LGI1, CASPR2, GABA_B_R, IgLON5, DPPX, GlyR_α_1, GABAR_α_1, GABAR_β_3, GABAR_γ_2, mGluR5, D2R, Neurexin-3α, and GAD65. According to the kit protocol the serum for analysis was diluted 1:10 with phosphate buffered saline (PBS), while the CSF was used without dilution. The diluted serum or original CSF was dripped onto the reaction area of the slides and placed in a humidity box for 1 h at room temperature. The slides were then washed with PBS followed by the addition of diluted FITC-goat anti human IgG and incubation for 30 min at room temperature in the humidity box. The slides were washed again with PBS, and then observed using a fluorescence microscope with a 20x objective lens.

Antibodies against neuronal intracellular antigens were detected in vitro by the dot immunobinding assay (DIBA) using the IFT Kit MT235-16 (Shanxi MYBiotech Co., Ltd. China). The IgG auto-antibodies included Hu, Yo, Ri, CV2, Ma2, Amphiphysin, Ma1, Tr, Zic4, PKCγ, SOX1, Recoverin, and Titin. According to the kit protocol the procedure included (1) Placing the test strip into the clean reaction well of a 12-well plate, followed by addition of serum or CSF samples to the reaction well and incubation on a shaking table at room temperature for 40 min; (2) washing the slides with PBS and incubation with a secondary antibody at room temperature for 30 min; (3) washing the slides with PBS, adding the diluted reaction substrate, and letting it stand at room temperature for 4 min. (4) drying the test strip at room temperature and analyzing the test result.

### Literature review and inclusion/exclusion criteria

We carried out a literature search of multiple databases including PubMed, EMBASE, Scopus, Web of Science, EBSCO, Google Scholar, and Wanfang, China National Knowledge Internet (CNKI) databases using the keywords “co-existing antibodies and autoimmune encephalitis” or “co-occurrence antibodies and autoimmune encephalitis” or “multiple antibodies and autoimmune encephalitis” or “GABA_B_R and Hu” or “GABA_B_R and NMDAR” or “GABA_B_R and SOX1” or “NMDAR and CASPR2”. The screening protocol was conducted according to the PRISMA workflow criteria. Inclusion criteria: The cases were required to have the co-existence of two or more kinds of anti-neuronal antibodies. Exclusion criteria: Cases with the anti-GFAP antibody or auto-antibodies associated with CNS demyelination (MOG, AQP4, and MBP) were excluded. The clinical characteristics, laboratory data, treatments, complications, and outcomes of all the patients were then summarized and reanalyzed.

## Results

### Clinical features of nine patients with multiple anti-neuronal antibodies

Nine AE patients (9/85, 10.6%) with co-existence of multiple anti-neuronal antibodies were identified from the 85 AE patients hospitalized in our department between August 2019 and February 2022. The male:female ratio was 4:5 with the age of onset ranging from 15 to 72 year (median 66 year). The clinical manifestations of the nine patients were consistent with encephalitis symptoms (Supplemental Table 1), and included disturbed consciousness, seizures, cognitive impairment, and psychiatric disorders. A pulmonary malignant tumor was suspected in two patients. Seven patients received immunotherapy (glucocorticoids, intravenous immunoglobulin (IVIG), plasmapheresis, or mycophenolate mofetil); while one patient was treated with acyclovir (1.5 g/day) and one patient refused any treatment. Collectively, six patients showed complete or partial neurological improvement after immunotherapy, one patient had a complete recovery after antiviral therapy alone, and two patients with suspected malignant tumors died during the follow-up period. Cerebral MRI revealed that four patients had abnormal lesions, mainly distributed in the temporo-parieto-occipital lobe, insular lobe, hippocampus, and basal ganglia. An electroencephalogram (EEG) was performed in eight patients, and showed an increase in slow waves in five patients, a mild abnormality in two patients, and epileptiform discharge in one patient. The auto-antibody panel showed six patients were positive for the NMDAR antibody (cases 1–5, and 9), with three patients having the CASPR2 antibody, two the GABA_B_R antibody, and one the GABA_B_R and GAD65 antibodies simultaneously. One patient (case 6) had co-existence of AMPAR2 and Hu antibodies, while two patients (cases 7 and 8) had LGI1 antibody co-existing with IGLON5 or CASPR2 antibody, respectively.

#### Patient 1

**Fig. 1:**
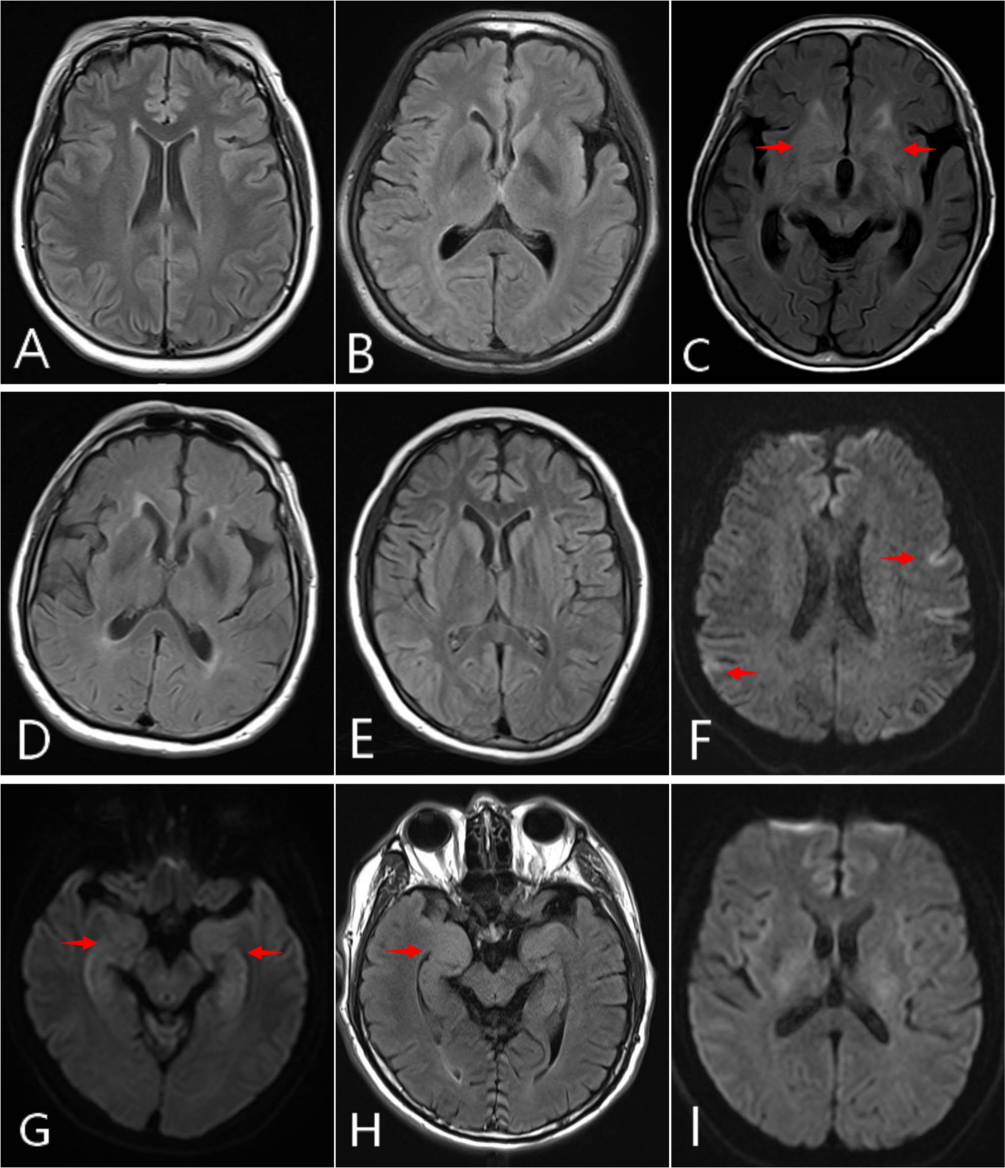
Cerebral MRI changes of 9 cases. Cerebral MRI revealed no abnormalities (**A** in patient 1; **B** in patient 2; **D** in patient 4; **E** in patient 5; **I** in patient 9). T2/Flair showed hyperintensities mostly involving the temporal lobe, parietal lobe, and basal ganglia area in patient 3 (**C**). Diffusion weighted imaging (DWI) showed hyperintensities involving the temporal and occipital cortex in patient 6 (**F**); DWI showed symmetrical hyperintensities in the bilateral temporal and insular cortex in patient 7 (**G**); and T2/flair showed swelling of the right hippocampus in patient 8 (**H**)

An adult woman (36 years old) was admitted for recurrent seizures over 20 years. The attacks occurred several times a year and lasted for 1–2 min. Other accompanying symptoms included a sleep disorder and cognitive impairment. Cranial MRI revealed no abnormalities (Fig. 1A). EEG indicated epileptic discharge in the left middle temporal area. CT scans of the chest and abdomen revealed no abnormalities, while serum tumor markers were within the normal range. Oxazepine and levetiracetam were administered for anticonvulsant therapy. After the identification of positive NMDAR and CASPR2 antibodies, high-dose methylprednisolone (0.5 g and 0.25 g, 3 d for each dose) was injected intravenously, followed by prednisone (60 mg daily) for 4 weeks, and then a gradual reduction in the dose by 5 mg every 2 weeks until the final withdrawal. Follow-up evaluation after 6 months showed that the symptoms had improved significantly and the patient was free of seizures.

#### Patient 2

An old woman (57 years old) presented with insomnia, a speech disorder, emotional lability, and confusion for 10 days. Cerebral MRI was normal (Fig. 1B) and EEG showed no abnormalities. CSF analysis showed the number of cells was 0/µL (reference 0–10/µL), protein level was 24 mg/dL (reference 15–45 mg/dL), and glucose level 3.5 mmol/L (reference 2.5–4.4 mmol/L). Chest and abdomen CT and serum tumor markers revealed no signs of a tumor. Following the detection of positive NMDAR and CASPR2 antibodies, intravenous acyclovir (1.5 g/day for 14 days) was administered. As antiviral drugs have a delayed effect when encephalitis is suspected an antiviral drug should be prophylactically used. However, subsequent to the diagnosis of autoimmune encephalitis, the patient and her family have refused the use of immunotherapy due to financial constraints and poor compliance. Interestingly, the patient felt no discomfort and her symptoms were completely relieved at the one-year follow-up.

#### Patient 3

An old woman (72 years old) presented with headache, limb weakness, and slow responses for 20 days and then developed disturbed consciousness and respiratory failure dependent on a ventilator. Neurological examination showed impaired memory, attention, and orientation. Cerebral MRI revealed multifocal T2/FLAIR hyperintensities mostly involving the temporal lobe, parietal lobe, and basal ganglia area (Fig. 1C). CSF analysis showed the number of cells was 30/µL, protein level of 77 mg/dL, and a normal glucose level. Chest and abdomen CT and serum tumor markers showed no sign of tumors. After identification of positive NMDAR and GABARβ3 antibodies, high-dose methylprednisolone was injected intravenously (0.5 g, 0.25 g, 3 days for each dose), accompanied by IVIG (0.4 g/kg for 5 days). Oral prednisone (50 mg daily) was administered for 8 weeks and then reduced gradually by 5 mg every 2 weeks until final withdrawal. The patient recovered gradually and was able to live independently. No tumors were found at the 6-month follow-up.

#### Patient 4

An old man (67 years old) presented with headache, dizziness, slurred speech, and slow responses for 3 days. Neurological examination revealed impaired cognition, attention, and orientation. Cerebral MRI showed no abnormalities (Fig. 1D), while CSF analysis revealed no pathological changes. Chest and abdominal CT scans identified multiple small pulmonary nodules, however, malignancy could not be confirmed due to the patient’s refusal to undergo a biopsy. Serum tumor markers showed no abnormalities. After identification of positive NMDAR and GABA_B_R antibodies, dexamethasone was injected intravenously(0.5 g, 0.25 g, 3 days for each dose), accompanied by IVIG (0.4 g/kg for 5 days). Oral prednisone (45 mg daily) was administered for 8 weeks, and then reduced gradually by 5 mg every 2 weeks until final withdrawal. The patient’s symptoms were greatly improved after immunological therapy. The multiple pulmonary nodules did not show obvious malignant transformation after 6 months.

#### Patient 5

A young girl (15 years old) was admitted with depression, severe insomnia, visual and acoustic hallucinations, and a suicide attempt within the past 15 days, and then developed disturbed consciousness and recurrent seizures. Cerebral MRI showed no abnormal findings (Fig. 1E). An EEG indicated a diffuse slow wave, while CSF analysis showed no abnormalities. A chest and abdomen CT showed multiple enlarged lymph nodes in the mediastinum, bilateral axilla, retroperitoneum, and bilateral inguinal, which a lymph node biopsy confirmed was lymphadenitis. After identification of positive NMDAR and CASPR2 antibodies, the patient was treated sequentially with plasmapheresis, intravenous high-dose methylprednisolone (1.0 g, 0.5 g, 0.25 g, 3 days for each dose), IVIG (0.4 g/kg for 5 days), and oral mycophenolate mofetil (0.5 g, 2 times daily). The patient’s symptoms had improved gradually and no tumors were found at the one-year follow-up.

#### Patient 6

**Fig. 2:**
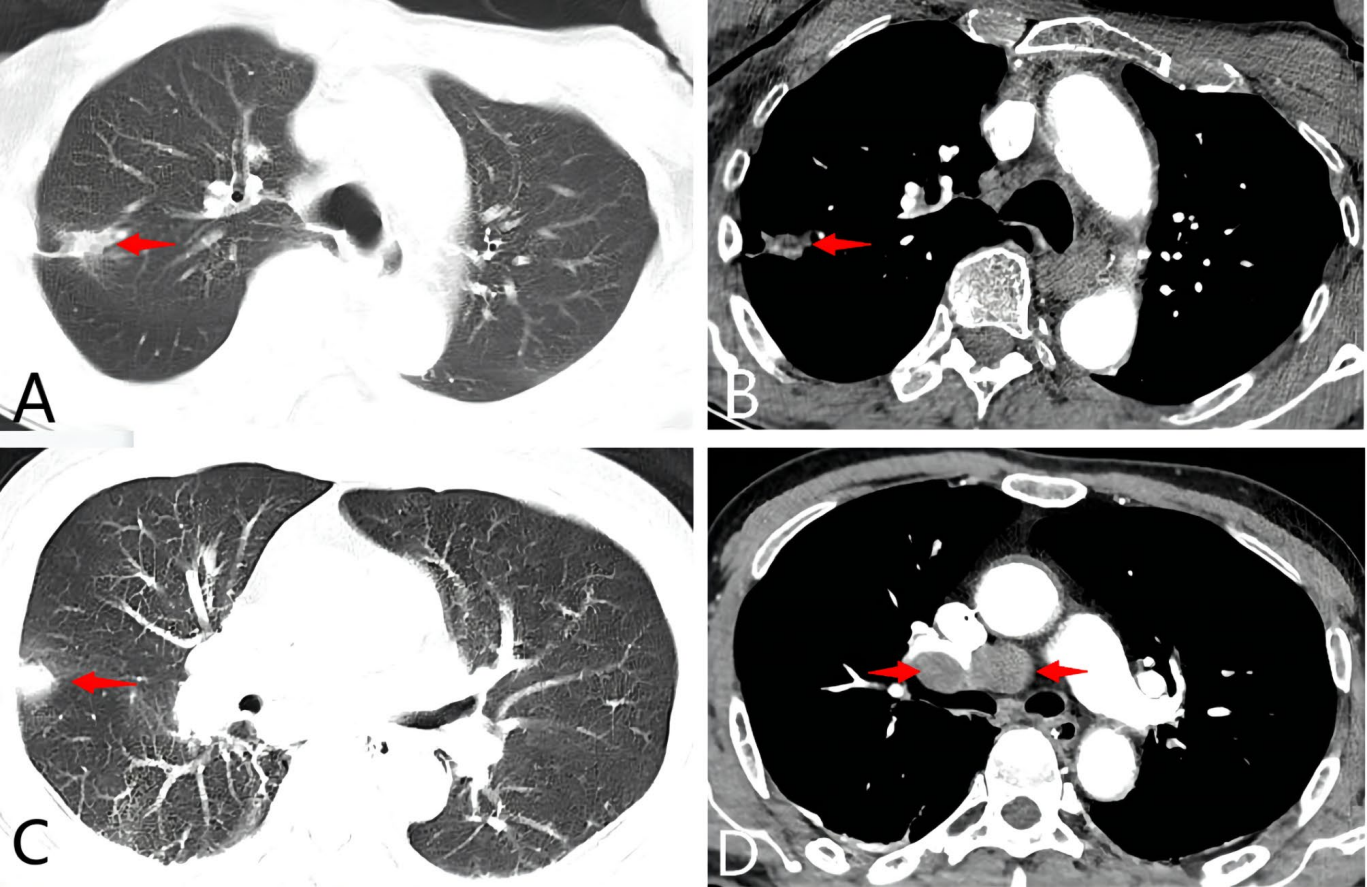
Enhanced chest CT changge of partial patients. Enhanced chest CT showed a nodule in the upper right lung in patient 6 (**A**, **B**); enhanced chest CT showed bilateral pulmonary multiple nodules in patient 9 (**C**), with multiple markedly enlarged lymph nodes in the right hilum (**D**)

An old man (71 years old) presented with cognitive impairment for half a month that progressed rapidly to a speech disorder, cognitive impairment, and confusion. Cerebral MRI revealed multifocal T2/FLAIR and DWI hyperintensities involving the left temporal and right occipital cortex (Fig. 1F). CSF analysis showed the number of cells was 1/µL, a protein level of 51 mg/dL, and a normal glucose level. An enhanced chest CT revealed a nodule in the upper right lung, which was suspected of being lung cancer (Fig. 2A, B). However, the patient and his families refused further evaluations and treatments. The patient was identified with positive AMPAR2 and Hu antibodies, and died four months after discharge.

#### Patient 7

An old woman (68 years old) presented with dizziness, a slow response, memory impairment, and a sleep disorder for half a month. Cerebral MRI revealed hyperintensities on T2/FLAIR involving the bilateral temporal and insular cortex (Fig. 1G), while CSF analysis showed no abnormalities. A chest and abdomen CT and serum tumor markers showed no signs of tumor. After identification of positive LGI1 and IGLON5 antibodies, the patient was treated with intravenous high-dose methylprednisolone (1.0 g, 0.5 g, 0.25 g, 3 days for each dose), IVIG (0.4 g/kg for 5 days), and oral mycophenolate mofetil (0.5 g, 2 times daily). Oral prednisone (60 mg daily) was administered for 4 weeks, and then reduced gradually by 5 mg every 2 weeks until the final withdrawal. The patient’s symptoms had improved gradually at the one-year follow-up.

#### Patient 8

An old man (66 years old) presented with memory decline, a speech disorder, and confusion for ten days, and then developed recurrent seizures, auditory hallucinations, and cognitive impairment. Neurological examination revealed cognitive impairment, a slow response, and a positive Babinski’s sign. Cerebral MRI revealed T2/FLAIR hyperintensities involving the right hippocampus and brain stem (Fig. 1H), while CSF analysis showed the number of cells was 2/µL, a protein level of 56 mg/dL, and a nomal glucose level. Chest and abdomen CT and serum tumor markers revealed no signs of tumor. After identification of positive LGI1 and CASPR2 antibodies, high-dose methylprednisolone (1.0 g, 0.5 g, 0.25 g, 3 days for each dosage) and IVIG (0.4 g/kg for 5 days) were administered simultaneously. During the 6 months of follow-up, the patient’s cognitive function improved and he was free of seizures.

#### Patient 9

An old man (65 years old) was admitted with recurrent seizures for one day that then developed rapidly to coma and severe respiratory insufficiency. Cerebral MRI showed no abnormalities (Fig. 1I). CSF analysis showed the number of cells was 4/µL, a protein level of 95 mg/dL, and a normal glucose level. An enhanced chest CT revealed multiple nodules in both lungs (Fig. 2C) and multiple markedly enlarged lymph nodes in the right hilum and mediastinum (Fig. 2D), which were considered to probably be malignant lesions. After identification of positive NMDAR, GABA_B_R, and GAD65 antibodies, high-dose methylprednisolone (1.0 g, 0.5 g, 0.25 g, 3 days for each dosage) and IVIG (0.4 g/kg for 5 days) were administered simultaneously. No significant improvement was observed, so the family preferred the patient received hospice care rather than further pathological biopsy and treatments. The patient died three days after being discharged.

### Literature summary

#### Retrieving the record

**Fig. 3:**
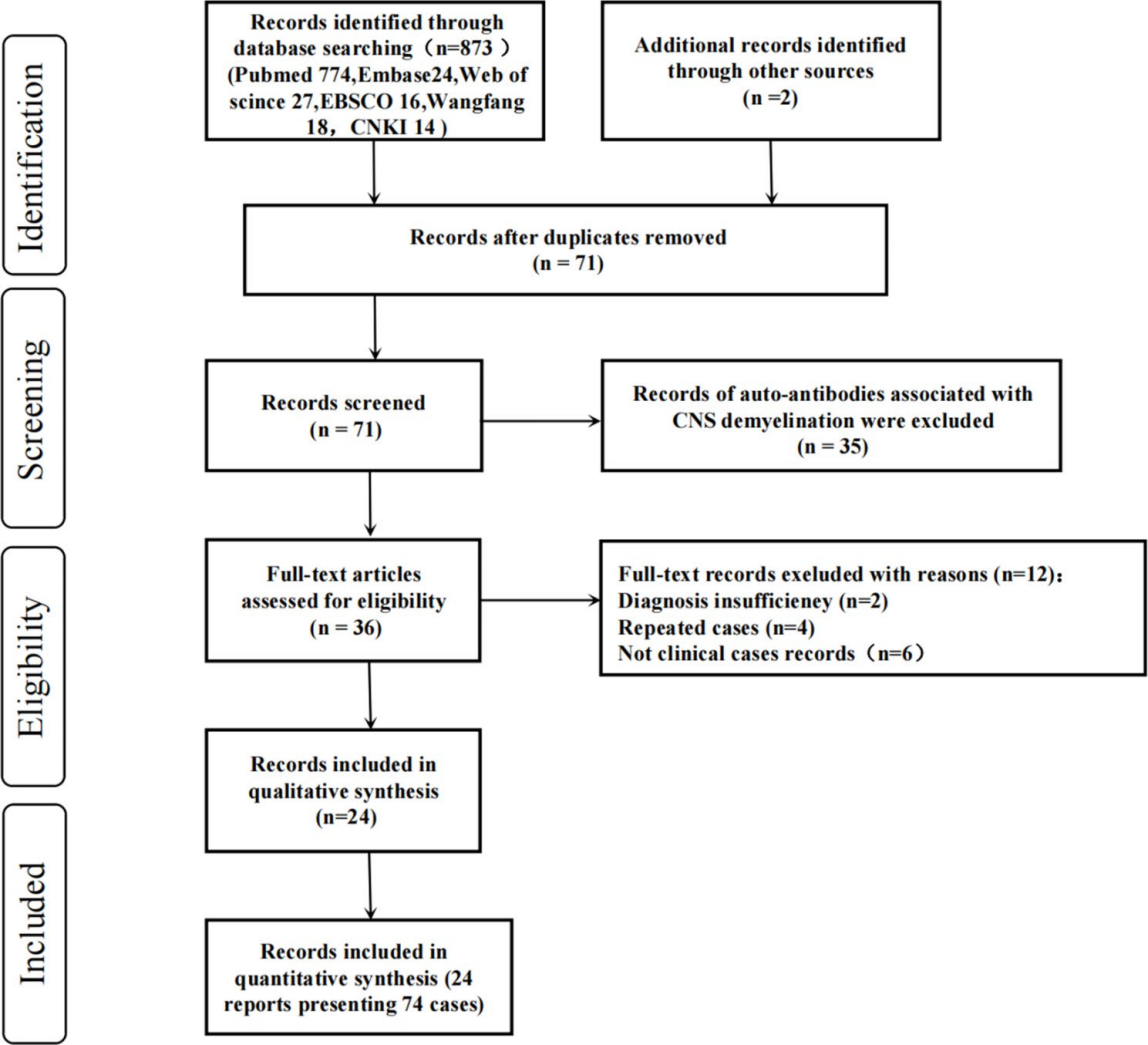
Flow diagram of studies selection process. In total, 873 records in the multiple databases were identified by the keywords. After removing duplicated records, 71 of the 873 records were screened for autoimmune encephalitis (AE) with co-existence of multiple anti-neuronal antibodies. Of these 71 records, 35 were excluded due to the presence of auto-antibodies associated with CNS demyelination. Twelve of the remaining 36 records were excluded due to diagnosis ineligibility with evidence insufficiency (n = 2), repeated report (n = 4) and no records of clinical cases (n = 6). Finally, 24 records including 74 cases qualified for review of clinical data on AE with co-existence of multiple anti-neuronal antibodies

The enrollment procedure used in this study is shown in Fig. 3. The selected keywords in the multiple databases identified a total of 873 records. After removing the duplicated records, 71 of the 873 records were screened for AE cases with co-existence of multiple anti-neuronal antibodies. Of these 71 records, 35 were excluded due to the presence of auto-antibodies associated with CNS demyelination, while 12 records were excluded due to diagnosis ineligibility with evidence insufficiency (n = 2), repeated reports (n = 4), or non-clinical case records (n = 6). Finally, 24 records [2–25] including 74 cases qualified for review of clinical data on AE with co-existence of multiple anti-neuronal antibodies.

#### Clinical summary

A summary of the clinical data of the 74 cases selected from the literature and the 9 cases described earlier is shown in Supplemental Table 1. Seventy-one cases from mainland China and 12 cases from outside of China. Of the 83 patients, 52 (62.6%) were male patients. The median age was 59 years (interquartile range [IQR] 45–66 years; range 4–84 years). The clinical manifestations of all the cases were consistent with typical encephalitis symptoms. Among the 76 cases with available detailed clinical symptoms (Table 1), cognitive impairment was the most common clinical presentation (53/76, 69.7%), followed by seizure (47/76, 61.8%), psychiatric symptoms (24/76, 31.6%), disturbed consciousness (18/76, 23.7%), sleep disorder (11/76, 14.5%), peripheral nerve symptom (11/76, 14.5%), hallucinations (10/76, 13.1%), and cerebellar symptoms (7/76, 9.2%). In addition, Li et al. [1] reported a single case of CASPR2 encephalitis combined with the Tr antibody, who presented with ocular muscle palsy and chorea. Ren et al. [4] also reported a single case of AMPAR encephalitis combined with the Hu antibody, who presented with psychiatric disturbance and bulbar palsy, while Li H et al. [5] reported a patient with co-existence of GABA_B_ and CRMP5/CV2 antibodies, who presented with Lambert–Eaton myasthenic syndrome.

**Table 1:** Clinical characteristics of 83 cases of autoimmune encephalitis (AE) with co-existence of multiple anti-neuronal antibodies

Characteristics	Values
**Sex, n(%)**	
Male	52(62.6%)
Female	31(37.4%)
Age, y, median (range)	59(4–84)
<20, n(%)	8(9.6%)
20–40, n(%)	9(10.8%)
40–60, n(%)	31(37.3%)
>60, n(%)	35(42.2%)
**Presenting symptom (n = 76)**	
Cognitive impairment, n(%)	53(69.7%)
Seizure, n(%)	47(61.8%)
Psychosis, n(%)	24(31.6%)
Disturbed consciousness, n(%)	18(23.7%)
Sleep disorder, n(%)	11(14.5%)
Peripheral nerve symptom, n(%)	11(14.5%)
Hallucination, n(%)	10(13.1%)
Cerebellar symptoms, n(%)	7(9.2%)
Tumor, n(%)	39(46.9%)
Lung cancer	28(33.7%)
Mediastinum tumor	3(3.6%)
Thymoma	2(2.4%)
Ovarian teratoma	2(2.4%)
Others^a^	4(4.8%)
**Outcomes, n(%)**	
Improvement	57(68.6%)
Died	26(31.3%)

^a^Hysteromyoma, breast cancer, bile duct cancer, and gastric cancer

#### Laboratory data (Table 2)

Forty-eight patients had an EEG examination with slow wave activity recorded in 28 cases, epileptic discharges in 12 cases, and mild or no abnormalities in 9 cases. Data on cerebral MRI were available in 66 cases, with 33 cases (33/66, 50.0%) having abnormal lesions. In brief, the high-intensity lesions were distributed mainly in the hippocampus (23/66, 34.8%), temporal lobes (18/66, 27.2%), basal ganglia (6/66, 9.1%), frontal lobe (6/66, 9.1%), occipital lobe (4/66, 6.0%), brainstem (4/66, 6.1%), and parietal lobe (2/66, 3.0%). These findings are consistent with the imaging feature of typical autoimmune encephalitis.

**Fig. 4:**
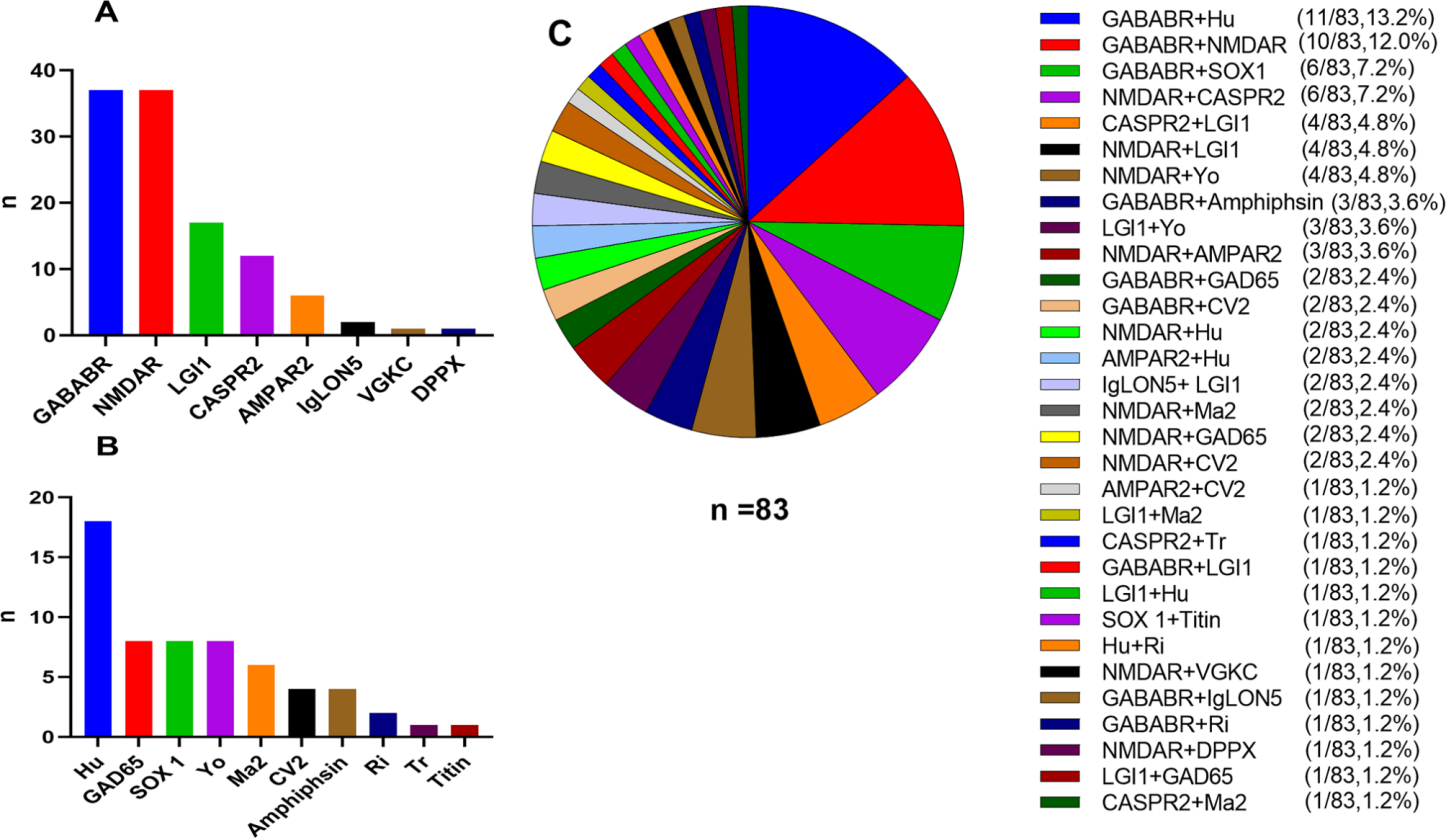
Distribution of anti-neuronal antibodies in 83 cases with autoimmune encephalitis (AE). Anti-neuronal surface antigen antibodies of 83 AE cases with co-existence of multiple anti-neuronal antibodies (**A**). Anti-neuronal intracellular antigen antibodies of 83 AE cases (**B**). Pie chart of autoantibody combinations in 83 cases (**C**)

The serum and CSF samples of all the patients were screened for the panel of anti-neuronal antibodies, which showed that 73 cases had co-existence of two types of anti-neuronal antibodies, 8 cases had three types of antibodies, and 2 cases had four types of antibodies. GABA_B_R and NMDAR antibodies were the two most common anti-neuronal surface antigen antibodies and were both found in 37 cases (37/83, 44.6%), followed by the LGI1 antibody in 17 cases (17/83, 20.5%), CASPR2 antibody in 12 cases (12/83, 14.5%), AMPAR2 antibody in 6 cases (6/83, 7.2%), IgLON5 antibody in 2 cases (2/83, 2.4%), VGKC antibody in one case (1/83, 1.2%), and DPPX antibody in one case (1/83, 1.2%). The Hu antibody (18/83, 21.7%) was the most common anti-neuronal intracellular antigen antibody, followed by GAD65, SOX1, and Yo antibodies each with 8 cases (8/83, 9.6%), Ma2 antibody in 6 cases (6/83, 7.2%), each CV2 and amphiphysin antibodies in 4 cases (4/83, 4.8%), Ri antibody in 2 cases (2/83, 2.4%), Tr antibody in one case (1/83, 1.2%), and Titin antibody in one case (1/83, 1.2%). The most common combination of auto-antibodies was GABA_B_R plus Hu in 11 patients (10/83, 13.2%), followed by GABA_B_R plus NMDAR in 10 patients (10/83, 12.0%), GABA_B_R plus SOX1 in 6 patients (6/83, 7.2%), and NMDAR plus CASPR2 in 6 patients (6/83, 7.2%). The detailed combinations are listed in Supplemental Table 1, Table 2; Fig. 4.

**Table 2:** Laboratory results and imaging of 83 autoimmune encephalitis (AE) cases with co-existence of multiple anti-neuronal antibodies

Variables	Positive number n(%)
**EEG (n = 48)**	
Slow activity	28(58.3%)
Epileptic activity	12(25.0%)
Mild or no abnormal	9(18.7%)
**Brain MRI (n = 66)**	
Frontal lobe	6(9.1%)
Temporal lobes	18(27.2%)
Parietal lobe	2(3.0%)
Occipital lobe	4(6.0%)
Hippocampus	23(34.8%)
Basal ganglia	6(9.1%)
Brainstem	4(6.1%)
No abnormalities	33(50.0%)
**Antibodies of AE**	
* Anti-neuronal surface antigen antibodies*	
GABA_B_R	37(44.6%)
NMDAR	37(44.6%)
LGI1	17(20.5%)
CASPR2	12(14.5%)
AMPAR	6(7.2%)
IgLON5	2(2.4%)
VGKC	1(1.2%)
DPPX	1(1.2%)
* Anti-neuronal intracellular antigen antibodies*	
Hu	18(21.7%)
GAD65	8(9.6%)
SOX 1	8(9.6%)
Yo	8(9.6%)
Ma2	6(7.2%)
CV2	4(4.8%)
Amphiphsin	4(4.8%)
Ri	2(2.4%)
Tr	1(1.2%)
Titin	1(1.2%)

#### Treatment and follow-up

Of the nine AE patients with multiple anti-neuronal antibodies treated at our center, except for two patients (P2 and P6) who declined treatment, both intravenous immunoglobulin (IVIG) and long-term corticosteroid were administered to 6 patients (P3, P4, P5, P7, P8, and P9), with two of these patients (P5 and P7) also receiving concurrent immunosuppressant therapy. The clinial outcomes showed complete recovery in 2 patients (P2 and P4), improvement in 5 patients (P1, P3, P5, P7, P8), and death in 2 patients (P6 and P9) both attributable to malignant neoplasms. The review of the prognostic data from all the cases showed that 39 of the 83 cases (46.9%) were confirmed or suspected to have tumors during treatment or follow-up, in which lung cancer was the most common (28/83, 33.7%), followed by mediastinal tumors (3/83, 3.6%), thymoma (2/83, 2.4%), ovarian teratomas (2/83, 2.4%), and other tumors (4/83, 4.8%). Of the 83 cases, 58 received first-line immunotherapy (i.e. glucocorticoids, IVIG, or plasmapheresis) and tumor removal; 15 received second-line immunotherapy (i.e. rituximab, cyclophosphamide, or mycophenolate mofetil) after first-line immunotherapy; 3 received symptomatic drug therapy; 4 refused treatment; while 7 had no available treatment details. Partial or complete recovery was achieved in 57 cases (57/83, 68.6%). Twenty-six cases (26/83, 31.3%) died during the follow-up period, with 24 of these deaths (24/26, 92.3%) confirmed or suspected to be associated with tumors.

## Discussion

Despite the increasing number of patients diagnosed with AE, only a minuscule proportion has been reported with co-existence of multiple anti-neuronal antibodies. The majority of these reports are case reports or single-center retrospective studies. The current study, collected and summarized the clinical characteristics and laboratory data of 83 AE patients with multiple anti-neuronal antibodies either treated at our center or identified in literature review. We found that the co-existence of multiple anti-neuronal antibodies might lead to superimposition or variation of clinical symptoms, which have a complicated impact on exacerbation of clinical symptoms and disease progression. This worse prognosis may be associated with the coexistence of multiple anti-neuronal antibodies, the presence of anti-intraneuronal antibodies, and the occurrence of concomitant tumor. Therefore, it was necessary to screen the tumors systematically and continuously to ensure early detection or intervention of potential tumors as well as the development of individualized immunomodulatory therapy.

In 2016, 10 (1.8%) patients with co-existence of multiple anti-neuronal antibodies were identified by testing serum and CSF auto-antibodies in 531 patients with unexplained encephalitis [4]. In 2020, Qiu et al. [6] reported that six (4.5%) patients had multiple anti-neuronal antibodies in a study cohort of 134 patients with AE or neurological paraneoplastic syndromes. Li et al. [1] in 2021 reported that of 64 patients with AE, 7 (10.9%) had multiple anti-neuronal antibodies, a proportion similar to the summary result of 10.6% in the current study. In contrast, Höftberger et al. [7] in 2013 showed that 7 of 20 patients (35.0%) with GABA_B_R encephalitis had coexistence with other anti-neuronal antibodies, which was significantly higher than that reported by previous studies. This might indicate a higher rate of coexistence of multiple anti-neuronal antibodies in GABA_B_R encephalitis. Furthermore, it was also reported in that study that GABA_B_R and NMDAR antibodies were the most common (both 44.6%,) in AE patients with co-existence of multiple anti-neuronal antibodies [7]. Therefore, we speculate that the proportion of AE patients with co-existence of multiple anti-neuronal antibodies is approximately 10%, with GABA_B_R and NMDAR being the most common positive antibodies in these patients. However, this proportion needs to be confirmed in future large-scale studies.

According to the classical paradigm of AE, a specific anti-neuronal antibody in an AE patient should correspond to distinct clinical features, an accompanied tumor, treatment responsiveness, and prognosis [26]. However, in clinical practice most specific autoantibody-related AEs usually present with considerable heterogeneity of symptoms, making in difficult to determine the association between autoimmune antibodies and clinical symptoms. For example, the clinical features of NMDAR encephalitis usually include seizures, psychiatric and behavioral abnormalities, autonomic dysfunction, and central hypoventilation [1]. Six of the nine patients in our study were positive for NMDAR antibody, with all of these patients presenting with some clinical features of NMDAR encephalitis, but also having some clinical features that were inconsistent with the condition. For example, patient 1 presented with a sleep disorder which is one of the clinical features in CASPR2 encephalitis, while patient 4 presented mainly with headaches and slow responsiveness, but did not have seizures, mental and behavioral abnormalities, and other characteristic clinical manifestations of NMDAR. These variations in symptoms indicated that there might be some modifying factors that influenced the clinical presentations in these patients. It is also possible that the coexistence of multiple auto-antibodies may account for the complicated manifestations in these patients.

Each anti-neuronal antibody typically corresponds to a distinct set of clinical features. Co-existence of multiple auto-antibodies might cause an overlapping of clinical features, thereby complicating clinical symptoms. For example, Ren et al. [4] reported that a case with GABA_B_R and Hu antibodies presented with seizures and sensory neuropathy, while Liu et al. [8] reported that a patient with co-existence of LGI1 and NMDAR antibodies presented with face-arm dystonic epilepsy (FBDS), cognitive decline superimposing on hyponatremia and disturbed consciousness. However, the clinical characteristics of some AE patients with multiple anti-neuronal antibodies do not simply overlap with the autoantibody-related symptoms, with some typical clinical features being absent, while novel clinical manifestations may appear. Chung et al. [9] reported the co-existence of IgLON5 and GABA_B_R antibodies in an AE patient who presented predominantly with typical IgLON5-related symptoms, including a severe sleep disorder, gait instability, dysarthria, and recurrent visual and acoustic hallucinations, but without the distinctive features of GABA_B_R-related symptoms of limbic encephalitis. In addition, Xia et al. [10] described the co-existence of GABA_B_R and CV2 antibodies in a patient who presented with seizures, memory deficits, and visual hallucination, despite hallucinations not having been reported previously in AE patients with GABA_B_R or CV2 antibodies. Therefore, the clinical manifestations in these patients with co-existence of multiple auto-antibodies might be more complicated, making it difficult to determine which antibody is pathogenic or concomitant in these patients. Some studies have suggested that the predominant symptoms in patients with multiple anti-neuronal antibodies are associated closely with the CSF positive auto-antibody [27].

At present, there are several hypotheses regarding the co-existence of multiple anti-neuronal antibodies in AE patients, although the pathogenic mechanism is not fully understood [4]. First, AE patients frequently have some particular tumors, which might be associated with multiple anti-neuronal antibodies such as GABA_B_R, Hu, SOX1, and CV2 antibodies in SCLC, or LGI1 and AMPAR antibodies in thymoma. Second, some AE patients might simultaneously have several tumors which induce the production of multiple auto-antibodies. Third, an infection by a pathogen might be associated with co-existence of multiple anti-neuronal antibodies. For example, Zhu et al. [11] reported a case of a child who developed AE with co-existence of GABA_B_R and NMDAR antibodies after herpes simplex virus encephalitis, while Yang et al. [12] reported an AE patient with NMDAR and AMPAR antibodies following infection by the human herpesvirus 7 (HHV-7) and development of an ovarian teratoma. Therefore, an autoimmune response secondary to the release of antigens from damaged brain tissue after a viral infection might be responsible for the production of multiple auto-antibodies.

AE can be divided into intra-cellular or cell-surface neuronal antibodies encephalitis, according to the location of the antigens targeted by the antibodies. In addition, AE can be divided into paraneoplastic AE, or nonparaneoplastic AE, based on whether or not a tumor is present [28]. Antibodies against intraneuronal antigens commonly belong to classic paraneoplastic antibodies. For example, 90% of patients with the Hu antibody have been reported to have small cell lung cancer, while patients with the Yo antibody had a higher risk of breast or ovarian cancer, and patients with the Tr antibody are usually found with lymphomas, with the majority of all these patients being poorly responsive to immunotherapy [29]. In contrast, patients with encephalitis with anti-neuronal cell surface antibodies respond well to immunotherapy, despite this type of encephalitis also being associated with tumors. About 50% of patients with GABA_B_R encephalitis also had a tumor, of which small cell lung cancer was the most common [30]. McKay et al. [31] reported that 86.3% of patients with GABA_B_R encephalitis showed partial or complete recovery after first-line or second-line immunotherapy. In our study, 52 AE patients had co-existence of anti-neuronal cell surface antibodies and anti-intraneuronal antibodies, with 25 patients having tumors, and 21 patients dying during follow-up. We also showed that 29 patients with AE had coexistence with 2 types of anti-neuronal cell surface antibodies, 7 of which were accompanied by tumors, with 3 cases dying. Two AE patients with two types of anti-intraneuronal antibodies developed tumors and both died during follow-up. Taken together, these results indicate that the types of auto-antibodies and accompanying tumors may impact the treatment as well as the clinical prognosis of patients with multiple anti-neuronal antibodies. Due to the majority of these patients having tumors, the prognosis of patients with anti-intraneuronal antibodies therefore depends mainly on the efficacy of antineoplastic therapy.In addition, anti-intraneuronal antibodies primarily cause damage to neurons via cytotoxic T cells, which results in worse responses to immunotherapy and therefore shorter survival [13]. In the current study, the treatment of AE patients was determined by the different attending physicians and also the wishes of the patients resulting in heterogeneity of clinical treatment. Based on the experiences in our center and the guidelines of Chinese expert consensus [32], IVIG plus long-term corticosteroid therapy resules in a relatively good outcome in most AE patients without tumors. However, plasmapheresis, different immunosuppressants, and biological agents against molecular targets are sometimes required in AE patients who present with severe symptoms or have a poor response to a corticosteroid. Taken together, these results indicate that comprehensive anti-neuronal antibody detection and thorough tumor screening should be taken seriously in patients with AE, with individualized immunotherapy and anti-tumor therapy having the potential to improve their prognosis.

Notably, the number of coexisting anti-neuronal antibodies might be associated with the progression and prognosis of patients with AE. Gagnon et al. [14] reported a patient with encephalitis associated with GAD65 and GABA_B_R antibodies, who presented with refractory status epilepticus. Although aggressive treatments were given with multiple anticonvulsants and immunosuppressive drugs were administered, a good recovery was not achieved and relapse occurred again 21 months after the onset of symptoms. This outcome indicated that the superposition of the GAD65 antibody may lead to aggravation of clinical symptoms in GABA_B_R encephalitis. Kammeyer et al. [15] reported a patient with NMDAR encephalitis associated with GAD65, Ma1, and Ma2 antibodies, who presented with disturbed balance, diplopia, and rapid deterioration in dyspnea, hypoxia, and intermittent confusion disorder, and eventually died within one month. In the current study, 18 of 73 patients with co-existence of two types of anti-neuronal antibodies died during follow-up, while 6 of 8 patients with co-existence of three types of anti-neuronal antibodies and 2 patients with four types of anti-neuronal antibodies died during follow-up. Of the 10 patients with co-existence of more than two types of anti-neuronal antibodies, a total of 8 cases were confirmed to also have a tumors, indicating that more types of co-existing anti-neuronal antibodies increase the risk of tumor development and a poorer prognosis.

It is imperative to acknowledge the limitations of this study. First, it was a retrospective study, and therefore clinical data in some cases were incomplete which reduced the clinical significance of our findings. For example, comprehensive detection of paraneoplastic antibodies was not performed in some patients. In order to account for this partially missing data, we only made descriptive statements without statistical analysis. Second, the descriptions of some reported cases were incomplete, which may have resulted in bias of about some important clinical features and laboratory results. Therefore, a prospective study in multiple centers needs to be carried out in the future. Third, although a variety of retrieval methods were used in the multiple databases, the search strategy might not have identified all reported AE cases with co-existence of multiple anti-neuronal antibodies, and some studies in the literature may have been missed. However, 83 patients were summarized in the study, which we consider was a sufficient number to evaluate the clinical characteristics of AE with co-existence of multiple anti-neuronal antibodies.

## Conclusions

In summary, the co-existence of multiple anti-neuronal antibodies is a specific AE subgroup which is increasingly recognized in clinical practice. However, further investigation is required to determine the precise pathogenesis of AE associated with multiple anti-neuronal antibodies, and to elucidate individualized immunotherapy and the impact of co-existing antibodies on clinical manifestations in the patients. In the future, the development of animal models for anti-neuronal antibodies will significantly enhance our understanding of disease pathogenesis.

### Electronic supplementary material

Below is the link to the electronic supplementary material.

**Supplementary Material 1**: Clinical information of 83 cases of autoimmune encephalitis with co-existence of multiple anti-neuronal antibodies

## Data Availability

All relevant data are described within the paper. Deidentified data can be requested. Data can be requested by all interested researchers, who can be contacted via the corresponding author.
