# Supplementary material for: Clinical characteristics of autoimmune encephalitis with co-existence of multiple anti-neuronal antibodies
Source: BMC Neurol. 2024 Jan 2;24:1. doi: 10.1186/s12883-023-03514-x (PMC10759401; doi:10.1186/s12883-023-03514-x)
Supplement: Supplementary file 1 — Supplementary Material 1: Clinical information of 83 cases of autoimmune encephalitis with co-existence of multiple anti-neuronal antibodies [file 12883_2023_3514_MOESM1_ESM.docx]

Supplemental data

**Clinical** **characteristics of** **autoimmune encephalitis** **with** **co-existence of multiple anti-neuronal antibodies**

Yiyi Zhou^#^, Hao Chen^#^, Min Zhu, Menghua Li, Lianqun Wang, Zunchun Xie, Meihong Zhou, Xiaomu Wu, Daojun Hong

Department of Neurology, the First Affiliated Hospital of Nanchang University, Nanchang, China

# the two authors have equal contribution to the work

Correspondence:

Daojun Hong, E-mail: hongdaojun@hotmail.com

Department of Neurology, the First Affiliated Hospital of Nanchang University, Nanchang, 330006, China.

Tel:+86-791-8869-2511; Fax: +86-791-8869-2511

Supplemental table 1 Clinical information of 83 cases of autoimmune encephalitis with co-existence of multiple anti-neuronal antibodies.

| **Case** | **Report** | **Age(y)/sex** | **Symptoms** | **Anti-neuronal surface antigen antibodies** | | **Anti-neuronal intracellular antigen antibodies** | | **EEG** | **MRI distribution** | **Tumor** | **Treatment** | **Outcome** |
| --- | --- | --- | --- | --- | --- | --- | --- | --- | --- | --- | --- | --- |
|  |  |  |  | **Serum** | **CSF** | **Serum** | **CSF** |  |  |  |  |  |
| 1 | Current study | 36/F | Seizure， sleep disorder，forgetfulness | NMDAR（1:10）  CASPR2（1:10） | — | — | — | Epileptic discharges in left middle temporal | No abnormal | — | Antiepileptic,GC | Sporadic epilepsy |
| 2 | Current study | 57/F | headache，psychosis | NMDAR（1:10）  CASPR2（1:10） | — | — | — | mild abnormal | No abnormal | — | antiviral | Fully recovered |
| 3 | Current study | 72/F | headache，Limb weakness, consciousness disturbance | GABARβ3 （1:10） | NMDAR（1:10） | — | — | increase in θ wave | Temporal, parietal,  basal ganglia | — | GC, IVIG | Improvement |
| 4 | Current study | 67/M | Headache,  unresponsive | GABA_B_R（1:10） | GABA_B_R（1:10）NMDAR  (1:1) | — | — | α rhythm is slow down and generalized | No abnormal | — | GC, IVIG | Fully recovered |
| 5 | Current study | 15/F | Seizure,consciousness disturbance,  psychosis | NMDAR  (1:32)  CASPR2（1:10） | NMDAR  (1:10) | — | — | severe diffusion abnormality | No abnormal | — | Antiepileptic,PP, GC, IVIG, Immunosuppressant | Improvement |
| 6 | Current study | 71/M | cognitive impairment | AMPAR2  (1:100) | AMPAR2  (1:1000) | Hu(+) | Hu(+) | ND | Temporal,  Occipital | suspected lung cancer | Refused treatment | Died |
| 7 | Current study | 68/F | dizziness, slow reaction, damaged memory， sleep disorder | IGLON5  (1:100),  LGI1  (1:10) | IGLON5  (1:10),  LGI1  (1:10) | — | — | mild abnormal | Temporal lobes, insular cortex | — | GC, IVIG, Immunosuppressant | Improvement |
| 8 | Current study | 66/M | Seizure,dementia,hallucination | LGI1 (1:32)  CASPR2（1:32） | LGI1 (1:10) | — | — | increase in θ wave | Hippocampus | — | Antiepileptic,GC ,IVIG | Improvement |
| 9 | Current study | 65/M | Seizure,psychosis,consciousness disturbance | GABA_B_R（1:32） | GABA_B_R（1:10）,  NMDAR  (1:3.2) | GAD65  (1:350) | GAD65  (1:10) | diffuse slowing wave | No abnormal | Suspecte lung cancer | Antiepileptic,GC ,IVIG | Died |
| 10 | Ren et al^[1]^ | 62/M | Seizure,Forgetfulness,drowsiness,bucking | GABA_B_R | GABA_B_R | Hu(+) | — | ND | No abnormal | Lung cancer | GC, IVIG | Improvement |
| 11 | Ren et al^[1]^ | 61/M | Seizure,Forgetfulness,drowsiness | GABA_B_R | GABA_B_R | Hu(+) | — | ND | No abnormal | Lung cancer | GC, IVIG | Improvement |
| 12 | Ren et al^[1]^ | 59/M | Seizure,psychosis | GABA_B_R | GABA_B_R | Hu(+) | Hu(+) | ND | Hippocampus | Lung cancer | GC, IVIG | Improvement |
| 13 | Ren et al^[1]^ | 58/M | Psychosis,Forgetfulness,numbness of limbs | GABA_B_R | GABA_B_R | Hu(+) | Hu(+) | ND | Hippocampus | Lung cancer | GC, IVIG | Died |
| 14 | Ren et al^[1]^ | 61/M | Seizure, Forgetfulness,coma | GABA_B_R | GABA_B_R, NMDAR | Hu(+) | — | ND | ND | Lung cancer | GC, IVIG | Died |
| 15 | Ren et al^[1]^ | 40/F | Seizure, aypnia, myodynia, fibrillation | LGI1 ,  CASPR2 | LGI1 | — | — | ND | No abnormal | — | GC, IVIG | Improvement |
| 16 | Ren et al^[1]^ | 56/F | Seizure, Forgetfulness, drowsiness | LGI1 | LGI1 | Yo | Yo | ND | No abnormal | — | GC, IVIG | Improvement |
| 17 | Ren et al^[1]^ | 50/F | Forgetfulness,psychosis | AMPAR | AMPAR | CV2 | CV2 | ND | No abnormal | Thymoma | ND | Improvement |
| 18 | Ren et al^[1]^ | 51/F | Psychosis, bucking, dysphagia | AMPAR | AMPAR | Hu | — | ND | Cerebral cortex | Mediastinum tumor | ND | Died |
| 19 | Li et al^[2]^ | 59/M | Seizure, coma | GABA_B_R | GABA_B_R | Hu | GAD65 | θ background rhythm | No abnormal | Lung cancer | IVIG, Immunosuppressant | Died |
| 20 | Li et al^[2]^ | 61/M | Seizure | GABA_B_R | GABA_B_R, NMDAR | ND | ND | diffuse slowing wave | No abnormal | Lung cancer | IVIG | Died |
| 21 | Li et al^[2]^ | 60/M | Seizure, Psychosis, cognitive impairment | GABA_B_R | GABA_B_R | Hu | — | — | No abnormal | Mediastinum tumor | GC, Immunosuppressant | Died |
| 22 | Li et al^[2]^ | 44/M | consciousness disturbance, unresponsive | — | NMDAR | ND | Yo | Slow wave background rhythm | Frontal, parietal, temporal, insula, hippocampus, basal ganglia | — | GC | Improvement |
| 23 | Li et al^[2]^ | 51/M | cognitive impairment, hallucination | — | NMDAR | Hu | — | — | Frontal and temporal lobes, cingulate gyrus, hippocampus, basal ganglia, brain stem | — | GC, IVIG | Improvement |
| 24 | Li et al^[2]^ | 66/M | decline in memory | LGI1 | — | Ma2 | — | — | No abnormal | — | GC | Improvement |
| 25 | Li et al^[2]^ | 54/M | Chorea, hypsomnia, eye muscle paralysis, ataxia | CASPR2 | CASPR2 | Tr | Tr | — | No abnormal | — | GC, IVIG, Immunosuppressant | Improvement |
| 26 | Hang shuang et al^[3]^ | 68/M | Seizure, Psychosis | GABA_B_R | NMDAR, GABA_B_R | — | — | Slow wave background rhythm | Hippocampus | Lung cancer | GC | Died |
| 27 | Liu xiaoyan et al^[4]^ | 57/F | Seizure, consciousness disturbance | LGI1, NMDAR | — | — | — | ND | — | — | GC, IVIG | Improvement |
| 28 | Zhu hongmin et al^[5]^ | 7/M | Seizure, consciousness disturbance, psychosis | NMDAR, GABA_B_R | NMDAR, GABA_B_R | — | — | Epileptoid discharge, diffuse slowing wave | Frontal , temporal, insular, hippocampus, basal ganglia | — | GC, IVIG, Immunosuppressant | Improvement |
| 29 | Xie Y et al^[6]^ | 60/M | seizures, irritability, confusion, memory loss, insomnia , abnormal behavior | LGI1， GABA_B_R | — | — | — | Moderate difusion abnormality | No abnormal | — | GC | Improvement |
| 30 | Wang XJ et al^[7]^ | 67/F | Memory loss, cognitive impairment, aphemia | LGI1 | — | Hu | — | Localized abnormality | Frontal , temporal, parietal , occipital lobe | — | GC | Improvement |
| 31 | Wang XJ et al^[7]^ | 57/F | seizures, memory loss, dystonia, drowsiness | NMDAR, LGI1 | — | — | — | ND | No abnormal | Lung cancer | GC, IVIG | Improvement |
| 32 | Wang XJ et al^[7]^ | 84/F | seizures, memory loss, cognitive impairment | — | GABA_B_R | Hu | — | ND | Hippocampus, temporal | Lung cancer | IVIG | Died |
| 33 | Wang XJ et al^[7]^ | 55/M | sensory aphasia, memory loss, | — | NMDAR | Ma2 | — | — | Hippocampus, temporal, occipital lobe | — | ND | Improvement |
| 34 | Wang XJ et al^[7]^ | 60/F | seizures, memory loss, cognitive impairment | — | GABA_B_R | amphiphsin | — | difusion abnormality | Hippocampus, temporal | Lung cancer | GC | Died |
| 35 | Wang XJ et al^[7]^ | 67/M | seizures, memory loss, psychosis, hyponatremia | LGI1 | NMDAR, LGI1 | — | — | ND | ND | — | GC | Improvement |
| 36 | Wang XJ et al^[7]^ | 43/F | seizures, memory loss, psychosis | — | NMDAR | Yo | — | ND | Hippocampus, temporal | Hysteromyoma | GC | Improvement |
| 37 | Qiu ZD et al^[8]^ | 82/F | memory loss, psychosis | GABA_B_R | GABA_B_R | amphiphysin | amphiphysin | ND | Hippocampus, temporal | Breast cancer | Refused treatment | No change |
| 38 | Qiu ZD et al^[8]^ | 62/F | Numbness and weakness of limbs, | — | — | SOX 1, Titin | SOX 1 | ND | ND | Bile duct cancer | ND | Died |
| 39 | Qiu ZD et al^[8]^ | 72/M | seizures, memory loss, numbness and weakness of lower limb | GABA_B_R | GABA_B_R | Amphiphysin, Hu, GAD 65 | Amphiphysin, Hu, GAD 65 | ND | ND | Gastric cancer | Refused treatment | Died |
| 40 | Qiu ZD et al^[8]^ | 62/M | seizures, memory loss | LGI1 | LGI1 | Yo | Yo | ND | Hippocampus | Pulmonary nodules | IVIG | Improvement |
| 41 | Qiu ZD et al^[8]^ | 70/M | weakness, cognitive impairment | LGI1, CASPR2 | — | — | — | ND | ND | Thymoma | IVIG | Improvement |
| 42 | Qiu ZD et al^[8]^ | 62/M | numbness and weakness of lower limb | — | — | Hu, Ri | Hu, Ri | ND | ND | Lung cancer | GC | Died |
| 43 | Reyes N et al^[9]^ | 25/F | seizures, memory lapses, bizarre behavior, auditory hallucinations | NMDAR,  VGKC | NMDAR | — | — | electrographic focal seizures | ND | Mature ovarian teratoma | GC, IVIG | Improvement |
| 44 | Chung HY et al^[10]^ | 58/M | depressive episode, gait instability, sleep disorder, hallucinations | IgLON5, GABA_B_R | IgLON5 | — | — | No abnormal | No abnormal | — | PP, GC, Immunosuppressant | Improvement |
| 45 | Kammeyer R et al^[11]^ | 66/M | worsening balance, diplopia, confusion | — | NMDAR | GAD65 | Ma1, Ma2 | diffuse and intermixed slowing | Brainstem, temporal lobes, basal ganglia | Suspected lung tumor | PP, GC, Immunosuppressant | Died |
| 46 | Gagnon MM et al^[12]^ | 38/F | confused disoriented, myoclonus, nystagmus | — | GABA_B_R | — | GAD 65 | epileptiform discharges | Hippocampus | — | GC, IVIG Immunosuppressant | Improvement |
| 47 | Kim AE et al^[13]^ | 67/M | cognitive impairment , gait instability, spasticity | — | NMDAR | Hu, CV2 | Hu, CV2 | ND | Basal ganglia | — | PP, GC, IVIG Immunosuppressant | Died |
| 48 | Höftberger R et al^[14]^ | 44/M | Limbic encephalitis | ND | GABA_B_R,  NMDAR | — | — | ND | ND | — | GC, Immunosuppressant | Improvement |
| 49 | Höftberger R et al^[14]^ | 63/F | Status epilepticus | — | GABA_B_R | — | GAD 65 | epileptiform discharges | ND | — | ND | Died |
| 50 | Höftberger R et al^[14]^ | 60/M | Limbic encephalitis | GABA_B_R | GABA_B_R | — | SOX1 | ND | ND | small-cell lung cancer | Chemotherapy | Died |
| 51 | Höftberger R et al^[14]^ | 62/M | Limbic encephalitis | GABA_B_R | GABA_B_R | — | Ri | ND | ND | small-cell lung cancer | ND | Died |
| 52 | Höftberger R et al^[14]^ | 68/F | Limbic encephalitis | GABA_B_R | GABA_B_R | — | SOX1 | ND | ND | small-cell lung cancer | GC, IVIG | Died |
| 53 | Höftberger R et al^[14]^ | 74/M | Limbic encephalitis | GABA_B_R | ND | SOX1 | ND | ND | ND | small-cell lung cancer | ND | Died |
| 54 | Höftberger R et al^[14]^ | 77/M | Limbic encephalitis | GABA_B_R | GABA_B_R | ND | Amphiphysin | ND | ND | small-cell lung cancer | GC, IVIG | Died |
| 55 | Qi HC et al^[15]^ | 30/F | Seizures, psychosis, hyponatremia | NMDAR, GABA_B_R | NMDAR | — | — | diffuse slowing wave | No abnormal | — | GC, IVIG | Improvement |
| 56 | Qi HC et al^[15]^ | 43/F | Seizures, memory impairments | LGI1, CASPR2 | LGI1 | — | — | diffuse slowing wave | Hippocampus, occipital lobe | — | GC, IVIG | Improvement |
| 57 | Qin W et al^[16]^ | 59/F | Seizures,  memory deficit | GABA_B_R | GABA_B_R | SOX1 | — | epileptiform discharge | Hippocampus | small-cell lung cancer | IVIG | Improvement |
| 58 | Yang J et al^[17]^ | 36/F | Seizures, psychosis, memory impairments | NMDAR,  AMPAR | NMDAR,  AMPAR | — | — | diffused slow wave | Temporal , hippocampus | ovarian teratoma | Antiviral, GC, IVIG, Immunosuppressant | Improvement |
| 59 | Liu RX et al^[18]^ | 49/M | Seizures, memory deficit, consciousness disturbance | — | NMDAR, GABA_B_R | — | — | diffused slow wave | No abnormal | small-cell lung cancer | Antiepileptic,GC, IVIG, Immunosuppressant | Died |
| 60 | Tian S et al^[19]^ | 21/M | Seizures, ataxia | NMDAR, | NMDAR,  DPPX | — | — | epileptiform discharge, diffused slow wave | Cerebellar, brainstem | — | Antiepileptic,GC | Improvement |
| 61 | Fan XM et al^[20]^ | 61/M | Seizures, memory impairments | LGI1 | LGI1 | GAD 65 | GAD 65 | epileptiform discharge | Hippocampus | — | Antiepileptic, GC,IVIG | Improvement |
| 62 | Xia J et al^[21]^ | 46/M | Seizures, memory impairment, visual hallucinations,consciousness disturbance | GABA_B_R | GABA_B_R | CV2 | CV2 | epileptiform discharge | Temporal , hippocampus | — | Antiepileptic, GC,IVIG | Improvement |
| 63 | Li H et al^[22]^ | 56/M | seizure,  memory impairments, weakness, change in character | GABA_B_R | ND | CV2 | ND | No abnormal | No abnormal | small-cell lung cancer | Antiepileptic, GC,IVIG, Chemotherapy | Improvement |
| 64 | Gong S et al^[23]^ | 56/M | seizures，cognitive impairment | GABA_B_R | GABA_B_R | SOX1 | — | epileptiform discharge | hippocampus | small cell lung cancer | Antiepileptic, GC,IVIG | Improvement |
| 65 | Qiao S et al^[24]^ | 62/F | Impaired memory, unsteady walking | NMDAR | NMDAR | CV2 | — | diffuse slowing wave | No abnormal | — | GC, IVIG, Immunosuppressant | Improvement |
| 66 | Qiao S et al^[24]^ | 10/M | Speech impairment, impaired memory, seizures | NMDAR | NMDAR | — | CV2 | diffuse slowing wave, epileptiform discharge | No abnormal | — | GC, IVIG | Improvement |
| 67 | Qiao S et al^[24]^ | 46/M | Abnormal sensation, seizures | LGI1 | LGI1 | Yo | — | diffuse slowing wave | No abnormal | — | GC, IVIG | Improvement |
| 68 | Qiao S et al^[24]^ | 69/M | Impaired memory, seizures, consciousness disturbance | GABA_B_R | GABA_B_R | Hu | — | diffuse slowing wave | No abnormal | small-cell lung cancer | GC, IVIG | Died |
| 69 | Qiao S et al^[24]^ | 60/F | Memory impairment, psychosis, seizures, generalized weakness | GABA_B_R | GABA_B_R | Hu, Yo | — | epileptiform discharge | No abnormal | small-cell lung cancer | Refused treatment | Died |
| 70 | Qiao S et al^[24]^ | 46/M | Memory impairment, psychosis, abnormal sensation, hallucinations | NMDAR | — | GAD 65  SOX1 | GAD 65  SOX1 | ND | ND | small-cell lung cancer | No immunotherapy | Improvement |
| 71 | Qiao S et al^[24]^ | 59/F | Sleep disorders, seizures, memory impairment | CASPR2 | — | Ma2 | — | diffuse slowing wave | No abnormal | — | GC, IVIG | Improvement |
| 72 | Qiao S et al^[24]^ | 25/M | Memory impairment, seizures,sleep disturbance | NMDAR,  AMPA1,  AMPA2 | NMDAR,  AMPA1,  AMPA2 | Ma2 | — | diffuse slowing wave | Frontal，insula | — | GC, IVIG | Improvement |
| 73 | Qiao S et al^[24]^ | 73/M | headache, unsteady walking, hallucinations, psychosis, consciousness disturbance | GABA_B_R | GABA_B_R | Hu | Hu | No abnormal | Multiple foci of ischemia | mediastinal tumors | GC, IVIG | Improvement |
| 74 | Qiao S et al^[24]^ | 70/M | Memory loss, cognitive decline, psychosis | GABA_B_R,  AMPA1 | GABA_B_R,  AMPA1 | SOX1 | — | ND | hippocampus | small-cell lung cancer | GC, IVIG | Died |
| 75 | Qiao S et al^[24]^ | 18/M | Fever, abnormal mental behavior, psychosis | NMDAR | NMDAR | Yo | — | ND | ND | — | PP, GC, IVIG Immunosuppressant | Improvement |
| 76 | Qiao S et al^[24]^ | 71/F | Seizures, psychosis, consciousness disturbance | NMDAR | NMDAR | Yo | — | ND | Frontal | — | GC, IVIG | Improvement |
| 77 | Qiao S et al^[24]^ | 4/M | Seizures, personality changes, consciousness disturbance | NMDAR | NMDAR | Ma2 | — | diffuse slowing wave | No abnormal | — | GC, IVIG | Improvement |
| 78 | Qiao S et al^[24]^ | 48/M | seizures，memory loss, cognitive decline,hallucinations, personality changes | LGI1 | LGI1 | Yo | — | diffuse slowing wave | hippocampus | — | GC, IVIG | Improvement |
| 79 | Qiao S et al^[24]^ | 17/M | Headache, seizures, numbness in right limb | NMDAR,  CASPR2 | NMDAR,  CASPR2 | — | — | diffuse slowing wave | No abnormal | — | GC, IVIG | Improvement |
| 80 | Qiao S et al^[24]^ | 14/F | Impaired memory, seizures, consciousness disturbance | NMDAR,  CASPR2 | NMDAR | — | — | No abnormal | No abnormal | — | GC, IVIG | Improvement |
| 81 | Qiao S et al^[24]^ | 14/M | Headache, memory loss, pain in both lower limbs, paroxysmal involuntary movements of both lower limbs | NMDAR,  CASPR2 | NMDAR | — | — | No abnormal | White matter lesions | — | GC, IVIG | Improvement |
| 82 | Qiao S et al^[24]^ | 64/M | Memory impairment, seizures, FBDS | NMDAR,  LGI1 | NMDAR,  LGI1 | — | — | diffuse slowing wave | Ischemic focus in the right frontal lobe | — | GC, IVIG | Improvement |
| 83 | Qiao S et al^[24]^ | 22/F | Headache, memory impairment, involuntary movements | NMDAR, | NMDAR,  AMPA1 | — | — | No abnormal | Temporal, hippocampus, brain stem | — | GC, IVIG | Improvement |

CSF: cerebrospinal fliud; AE：autoimmune encephalitis；F: female; FBDS: faciobrachial dystonic seizure; GC: glucocorticoids; IVIG: intravenous human immunoglobulin; M: male; ND: no data. PP: Plasmapheresis;

**References**

[1] Ren HT, Yang XZ, Guang HZ, Gao XY, Peng B, Zhu YC, et al. Clinical analysis of autoimmune encephalitis with co-existence of multiple anti-neuronal antibodies. Chinese Journal of Neurology 2016, 49(01): 21-25.

[2] Li HH, Cheng XS, Feng LL, Sun C, Chang T. Clinical analysis of autoimmune encephalitis with co-existence of multiple anti-neuronal antibodies. Chinese Journal of Neurology 2021, 54(02): 92-98.

[3] Han S, Cao YT, Xu WH, Xia JY, Zhang CL, Cao J, et al. Autoimmune encephalitis with anti-GABAB-R antibody and anti-NMDA-R antibody: a case report and literature review. Journal of Apoplexy and Nervous Diseases 2018, 35(11): 1017-1018.

[4] Liu XY, Cheng Z, Wu GL, Peng T. A case of autoimmune encephalitis with positive for anti-LGI1 and anti-N-methyl-D-aspartate receptor antibodies. National Medical Journal of China 2020, (11): 871-872.

[5] Zhu HM, Sun D, Hu JS, Wu GF, Luo XQ, Liu ZS. Anti-NMDAR encephalitis and anti-GABABR encephalitis in a child after herpes simplex encephalitis. Chinese Journal of Neuromedicine 2020, (01): 73-74-75.

[6] Xie Y, Wen J, Zhao Z, Liu H, Xie N. Autoimmune encephalitis with coexistent LGI1 and GABABR1 antibodies: case report. BMC Neurol 2021, 21(1): 461.

[7] Wang XJ, Wang MH, Yu L, Peng T, Hu WT, Sun GF, et al. Clinical analysis of autoimmune encephalitis with multiple anti-neuronal antibodies. Chinese Journal of Practical Nervous Diseases 2020, 23(06): 491-496.

[8] Qiu ZD, Liu Z, Li DW, Song XD, Wang JS, Dong HQ. Clinical analysis of paraneoplastic neurological syndrome with co-existence of multiple anti-neuronal antibodies. Chinese Journal of Neuroimmunology and Neurology 2020, 27(04): 261-265.

[9] Reyes N, Prado MB, Turalde C, Fernandez M. Autoimmune encephalitis associated with two antibodies. Epilepsy Behav Case Rep 2018, 10: 44-46.

[10] Chung HY, Wickel J, Voss A, Ceanga M, Sell J, Witte OW, et al. Autoimmune encephalitis with anti-IgLON5 and anti-GABAB-receptor antibodies: A case report. Medicine (Baltimore) 2019, 98(20): e15706.

[11] Kammeyer R, Piquet AL. Multiple co-existing antibodies in autoimmune encephalitis: A case and review of the literature. J Neuroimmunol 2019, 337: 577084.

[12] Gagnon MM, Savard M, Mourabit Amari K. Refractory status epilepticus and autoimmune encephalitis with GABAAR and GAD65 antibodies: A case report. Seizure 2016, 37: 25-7.

[13] Kim AE, Kang P, Bucelli RC, Ferguson CJ, Schmidt RE, Varadhachary AS, et al. Autoimmune Encephalitis With Multiple Autoantibodies: A Diagnostic and Therapeutic Challenge. Neurologist 2018, 23(2): 55-59.

[14] Höftberger R, Titulaer MJ, Sabater L, Dome B, Rózsás A, Hegedus B, et al. Encephalitis and GABAB receptor antibodies: novel findings in a new case series of 20 patients. Neurology 2013, 81(17): 1500-6.

[15] Qi HC. Multi-anti-neuronal antibody-positive autoimmune encephalitis: 2 case reports and review of literature [D]. Guangxi: Guangxi Medical University ,2018.

[16] Qin W, Wang X, Yang J, Hu W. Coexistence of Anti-SOX1 and Anti-GABAB Receptor Antibodies with Autoimmune Encephalitis in Small Cell Lung Cancer: A Case Report. Clin Interv Aging 2020, 15: 171-175.

[17] Yang J, Wu P, Liu X, Xia H, Lai Z. Autoimmune Encephalitis With Multiple Auto-Antibodies With Concomitant Human Herpesvirus-7 and Ovarian Teratoma: A Case Report. Front Med (Lausanne) 2021, 8: 759559.

[18] Liu RX, Han XL, Lin X, Zhou YL, Zhao H, He JY, et al. A case of autoimmune encephalitis with multiple anti-neuronal antibodies associated with immune check-point inhibitors. Chinese Journal of Nervous and Mental Diseases 2022, 48(01): 48-50.

[19] Tian S, Zheng HQ, Liu P, Wu LX, Wu W. Autoimmune encephalitis with double positive anti-N-methyl-D-aspartate receptor and dipeptidyl-peptidase-like protein-6 antibodies: a case report. Chinese Journal of Neurology 2021, 54(03): 255-257.

[20] Fan XM, Liu SY, Huang L, Zhu YF, Zheng Q, Huang XH, et al. Anti-leucine-rich glioma-inactivated protein 1 and glutamic acid decarboxylase 65 antibodies double-positive autoimmune encephalitis complicated with vitiligo: a case report. Chinese Journal of Neurology 2020, 53(10): 810-813.

[21] Xia J, Yin X, Zhu M, Cao J, Song X. Autoimmune encephalitis positive for both anti-γ-aminobutyric acid B receptor and anticollapsin response-mediator protein 5 antibodies: A case report. Medicine (Baltimore) 2018, 97(3): e9574.

[22] Li H, Zhang A, Hao Y, Guan H, Lv Z. Coexistence of Lambert-Eaton myasthenic syndrome and autoimmune encephalitis with anti-CRMP5/CV2 and anti-GABAB receptor antibodies in small cell lung cancer: A case report. Medicine (Baltimore) 2018, 97(19): e0696.

[23] Gong S, Han Y, He E, Liu M, Fu X, Deng F. Coexistence of anti-SOX1 and anti-GABAB receptor antibodies with paraneoplastic limbic encephalitis presenting with seizures and memory impairment in small cell lung cancer: A case report. Front Immunol 2022, 13: 955170.

[24] Qiao S, Zhang SC, Wang ZH, Wang L, Zhang RR, Li HY, et al. Coexistence of multiple anti-neuronal antibodies in autoimmune encephalitis in China: A multi-center study. Front Immunol 2022, 13: 858766.

**
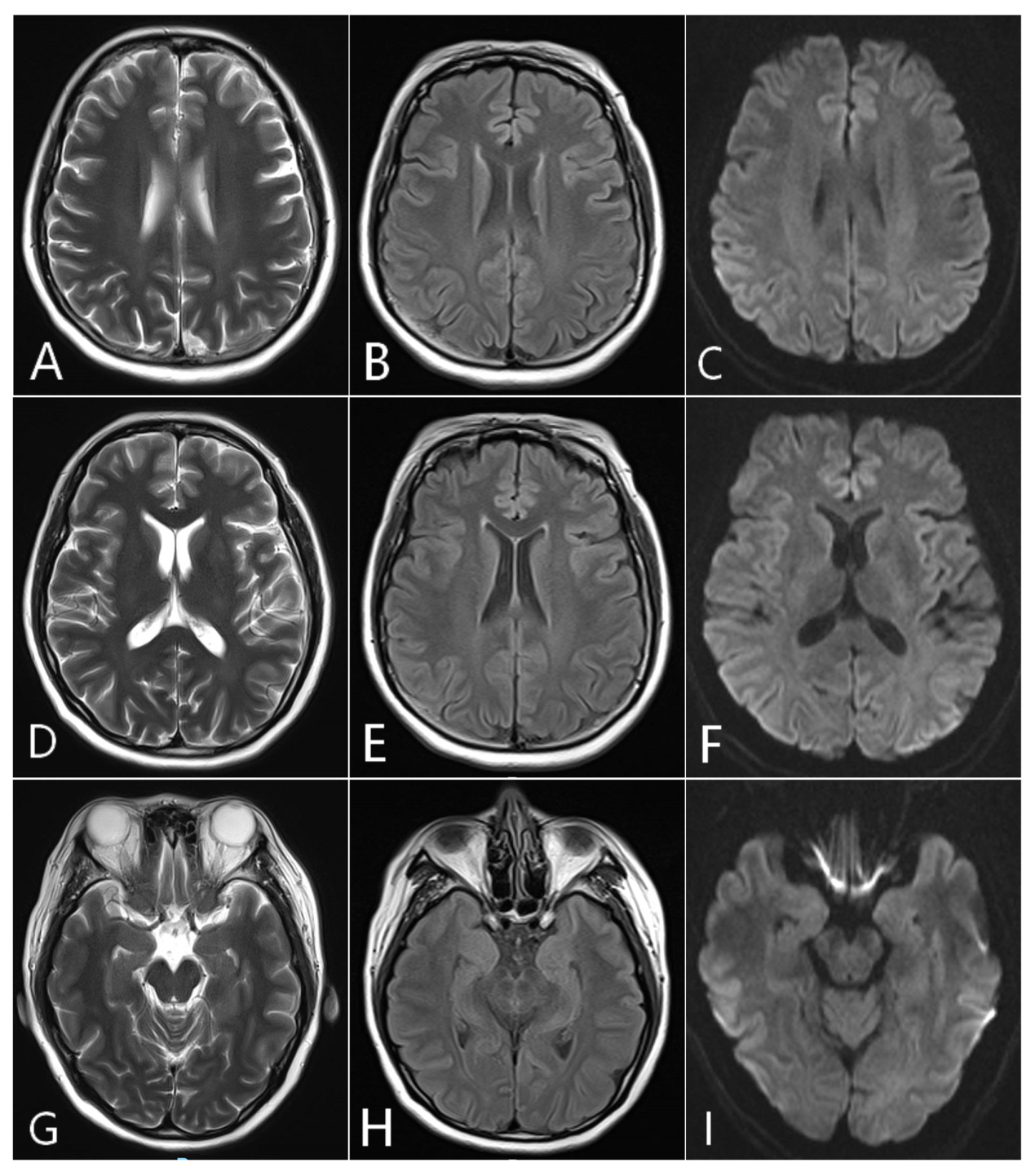
**

**Figure S1.** The cerebral MRI in patient 1 with positive NMDAR and CASPR2 antibodies.


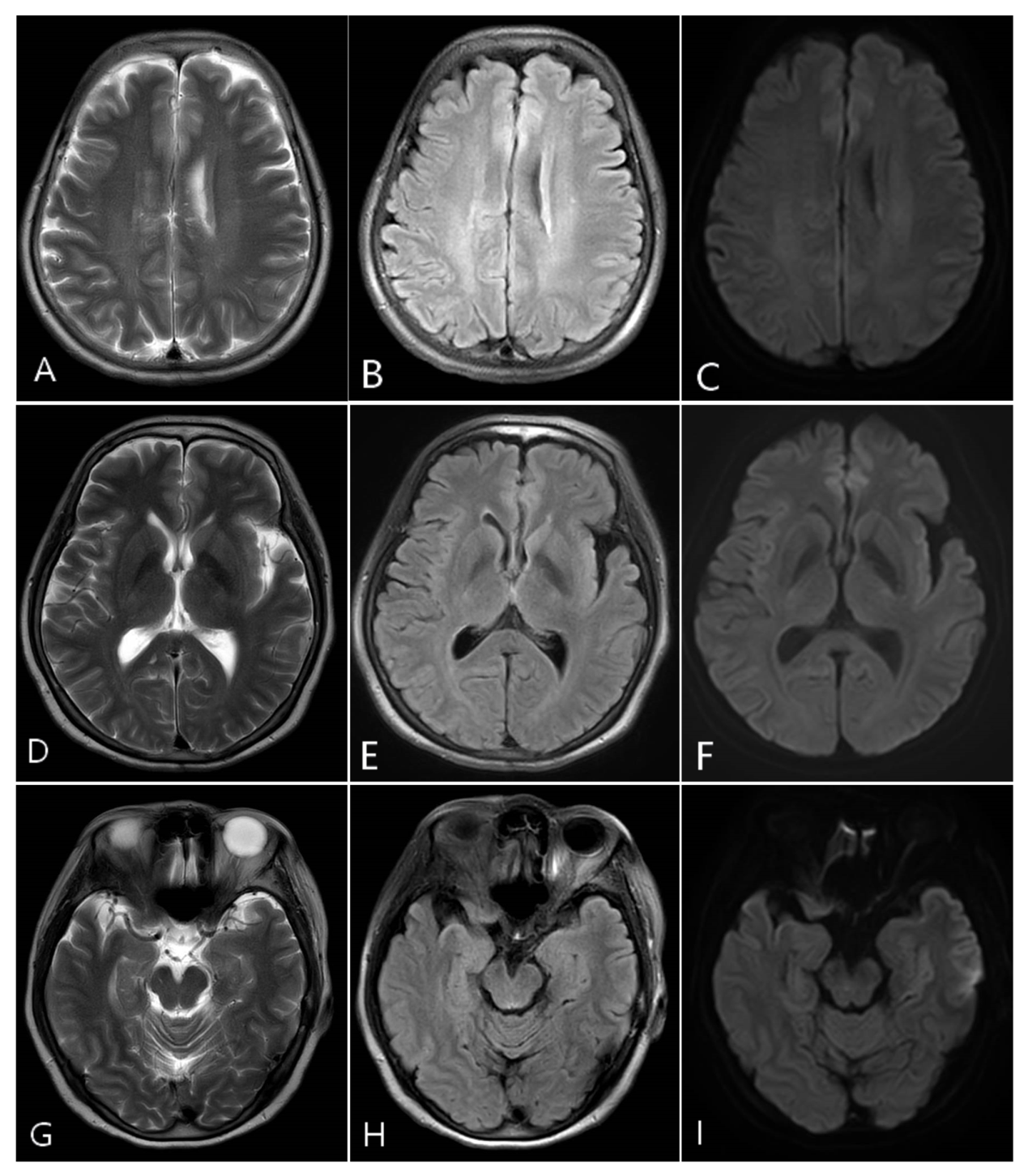


**Figure S2.** The cerebral MRI in patient 2 with positive NMDAR and CASPR2 antibodies.


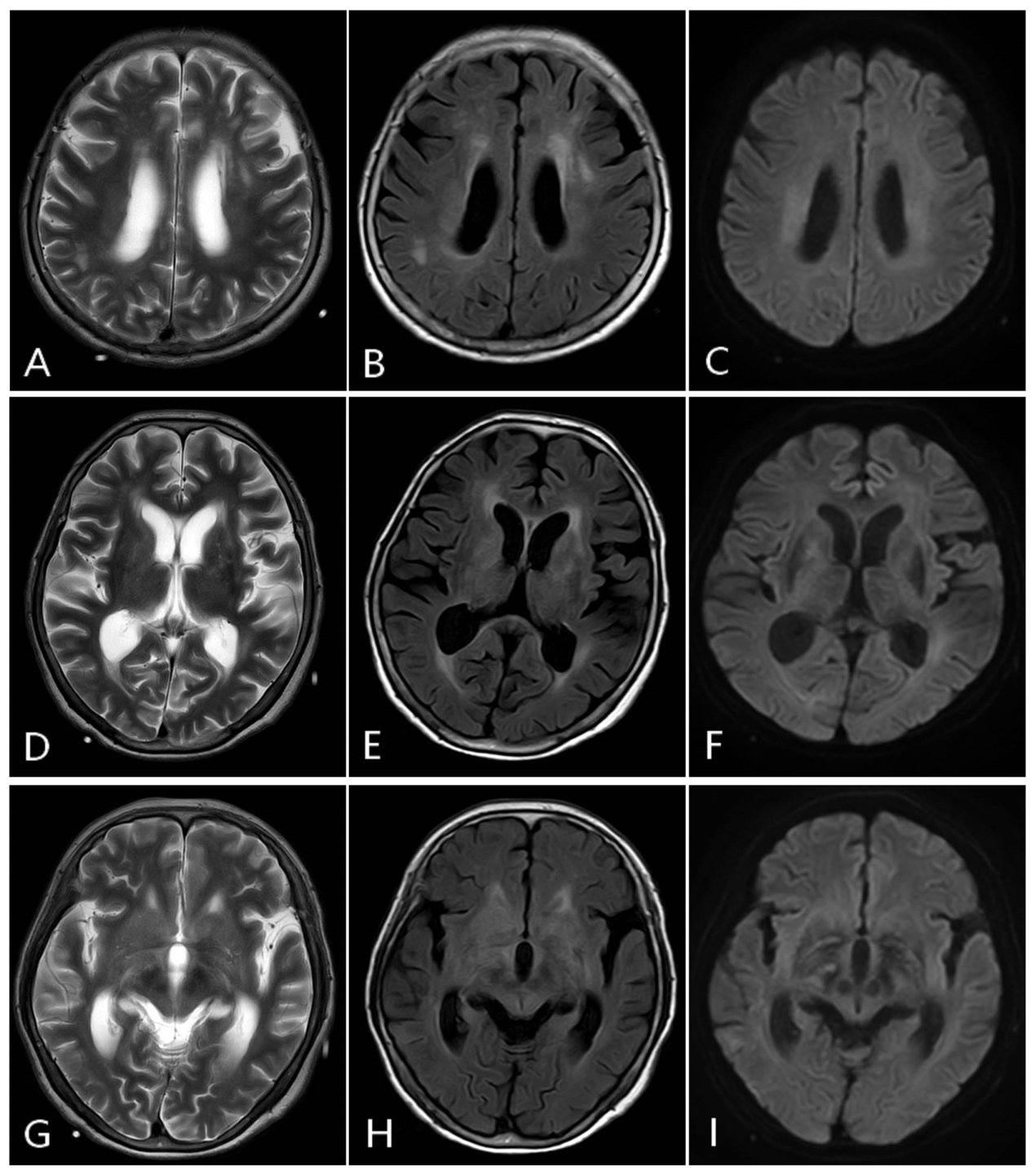


**Figure S3.** The cerebral MRI in patient 3 with positive NMDAR and CASPR2 antibodies.

**
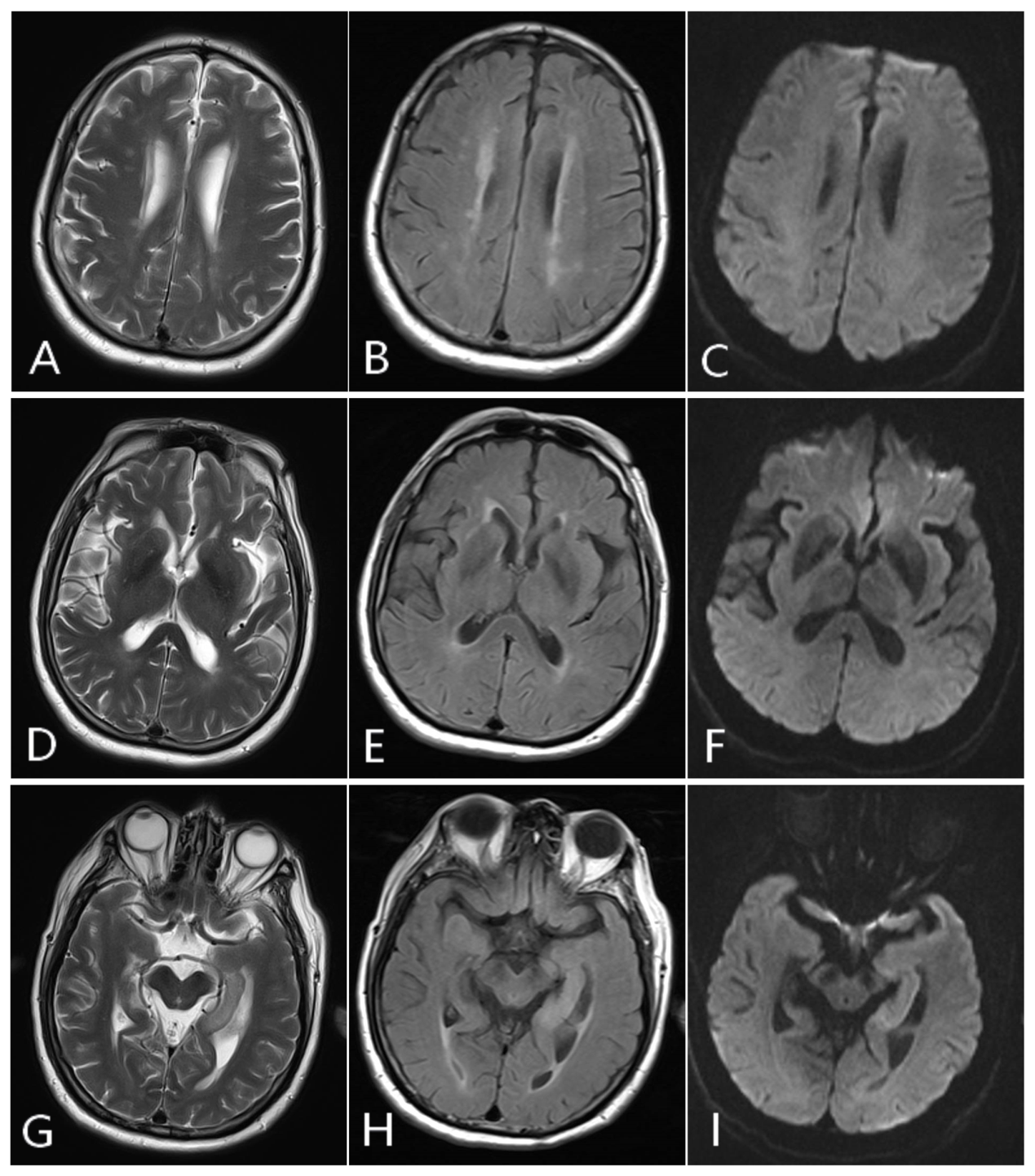
**

**Figure S4.** The cerebral MRI in patient 4 with positive NMDAR and GABA_B_R antibodies


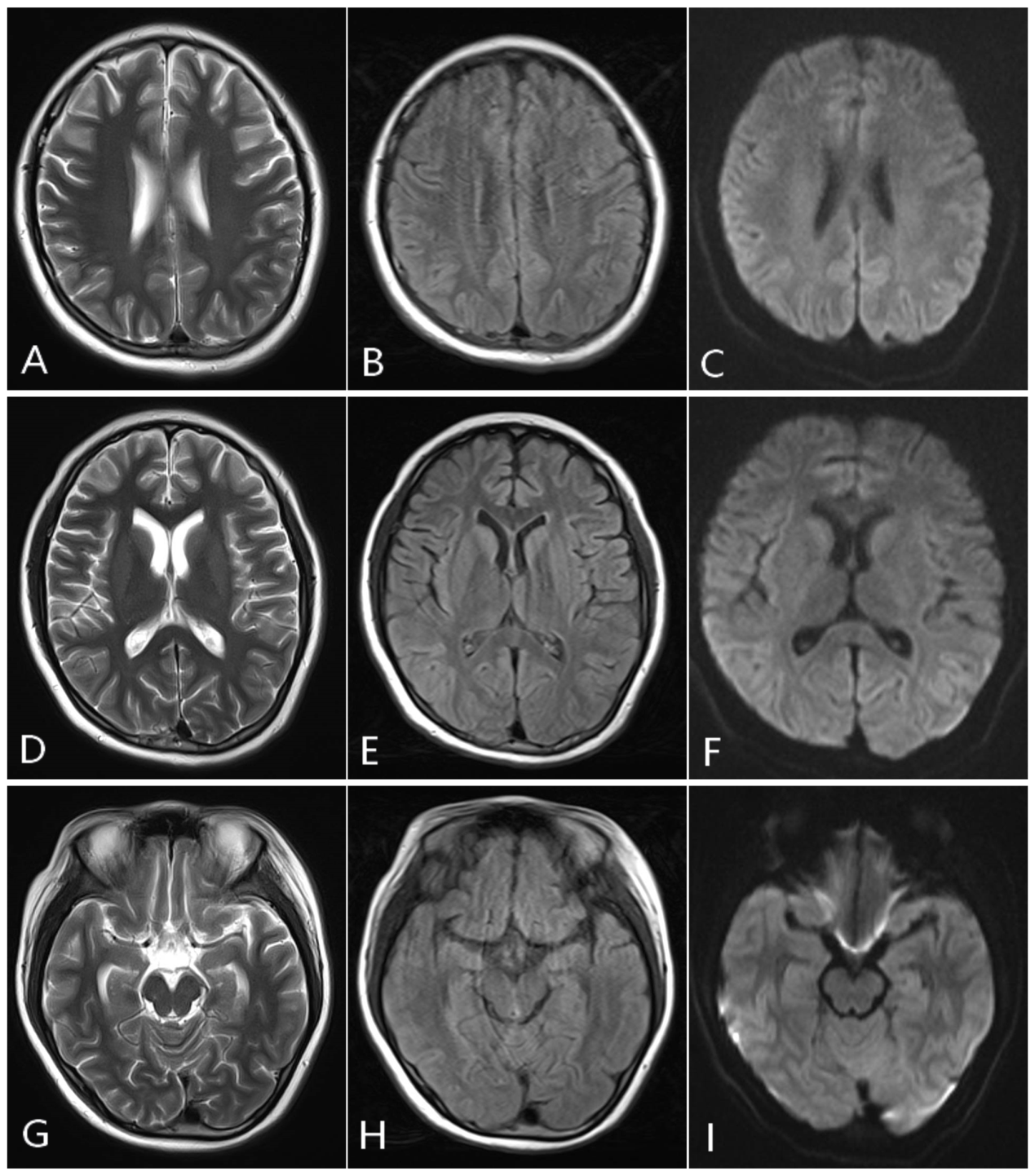


**Figure S5.** The cerebral MRI in patient 5 with positive NMDAR and GABA_B_R antibodies.


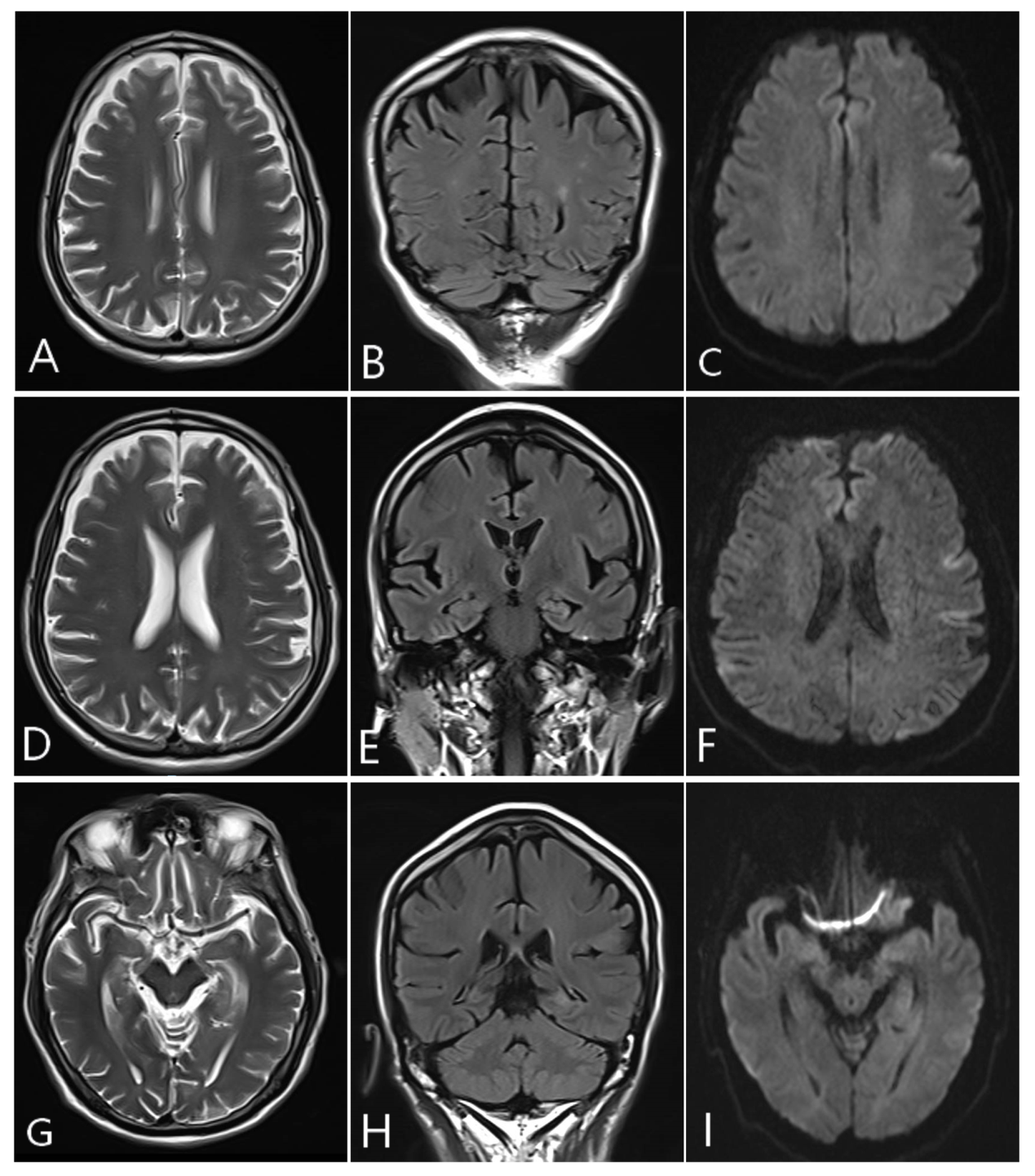


**Figure S6.** The cerebral MRI in patient 6 with positive AMPAR2 and Hu antibodies.


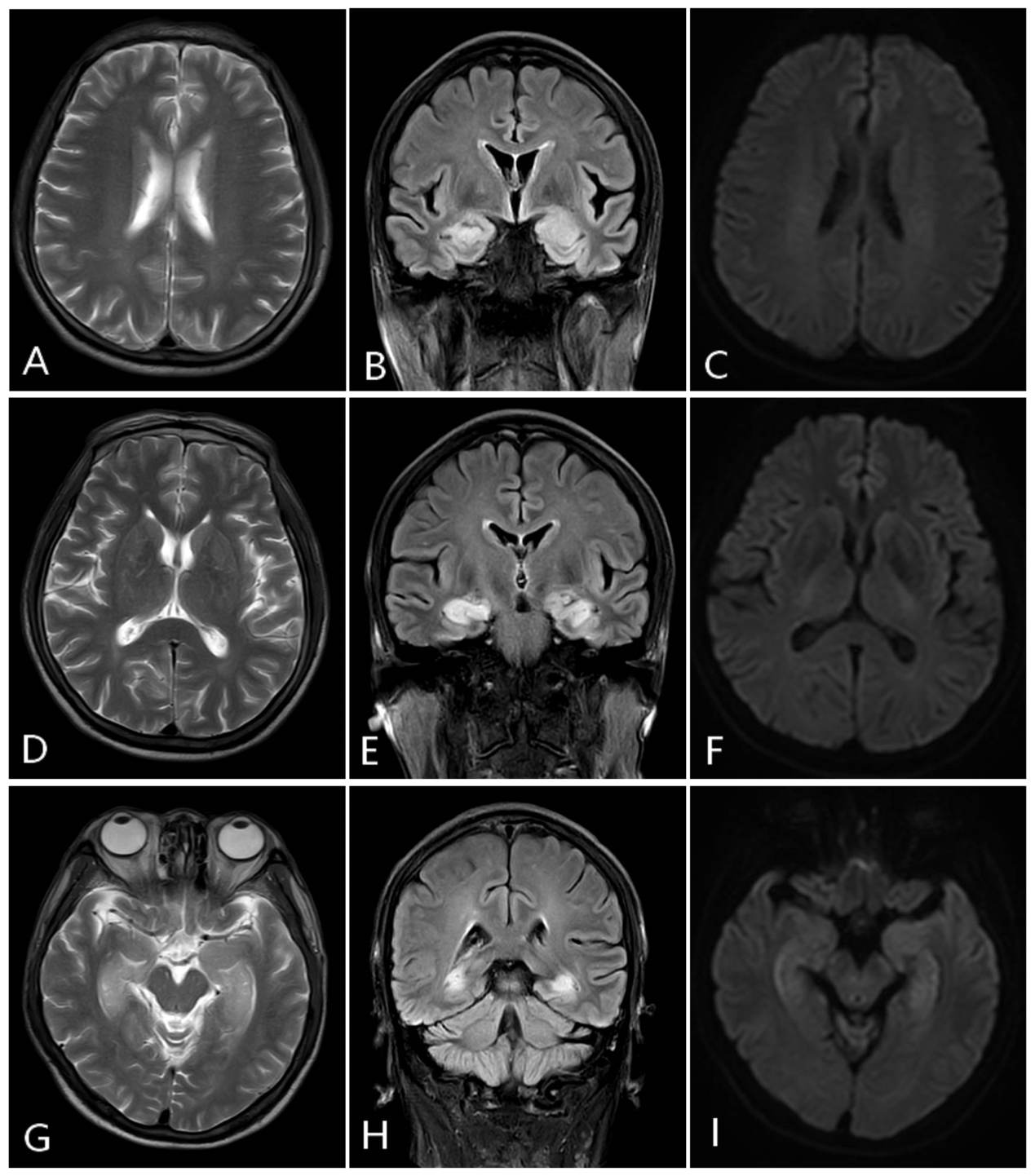


**Figure S7.** The cerebral MRI in patient 7 with positive LGI1 and IGLON5 antibodies.


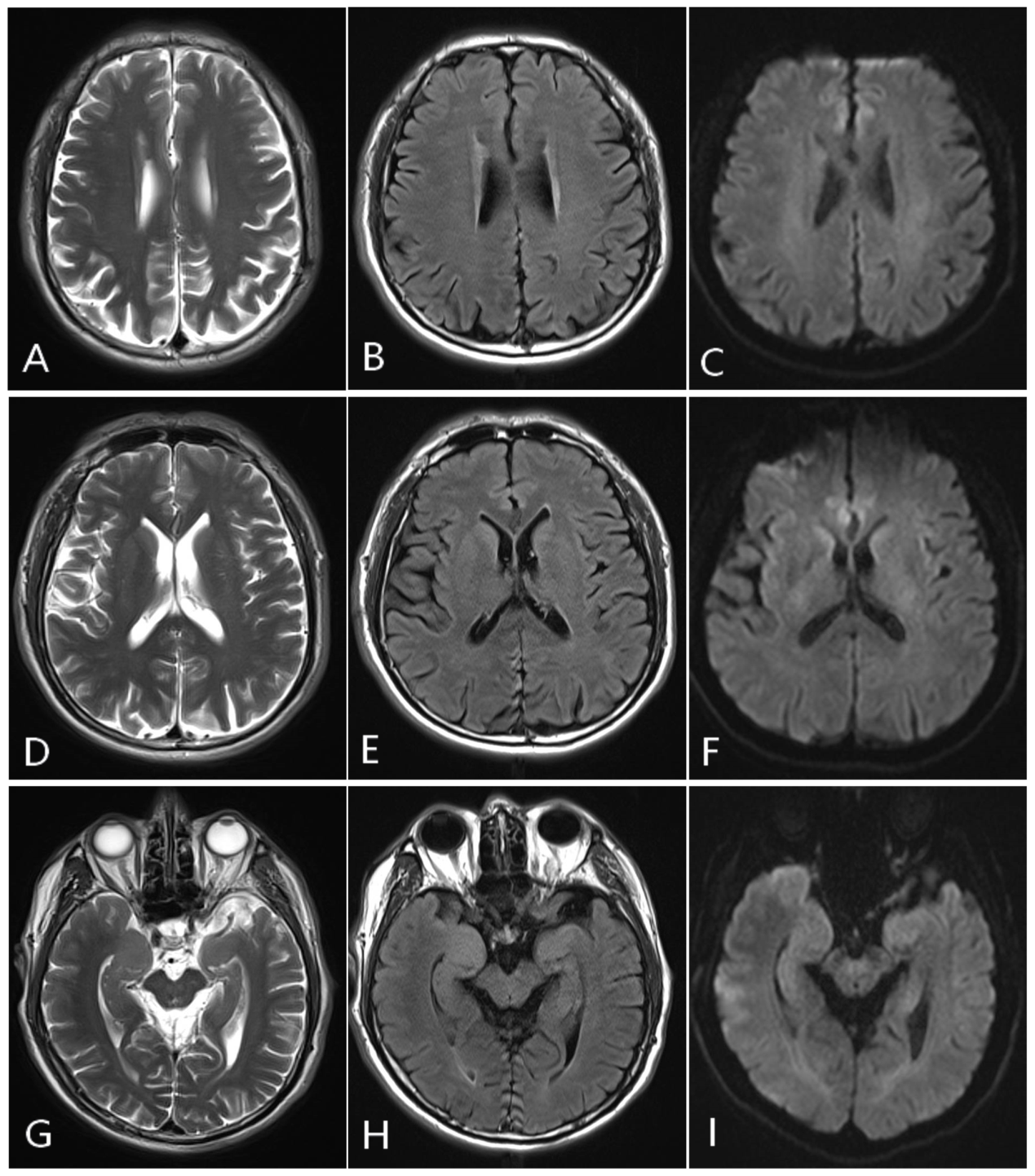


**Figure S8.** The cerebral MRI in patient 8 with positive LGI1 and CASPR2 antibodies.


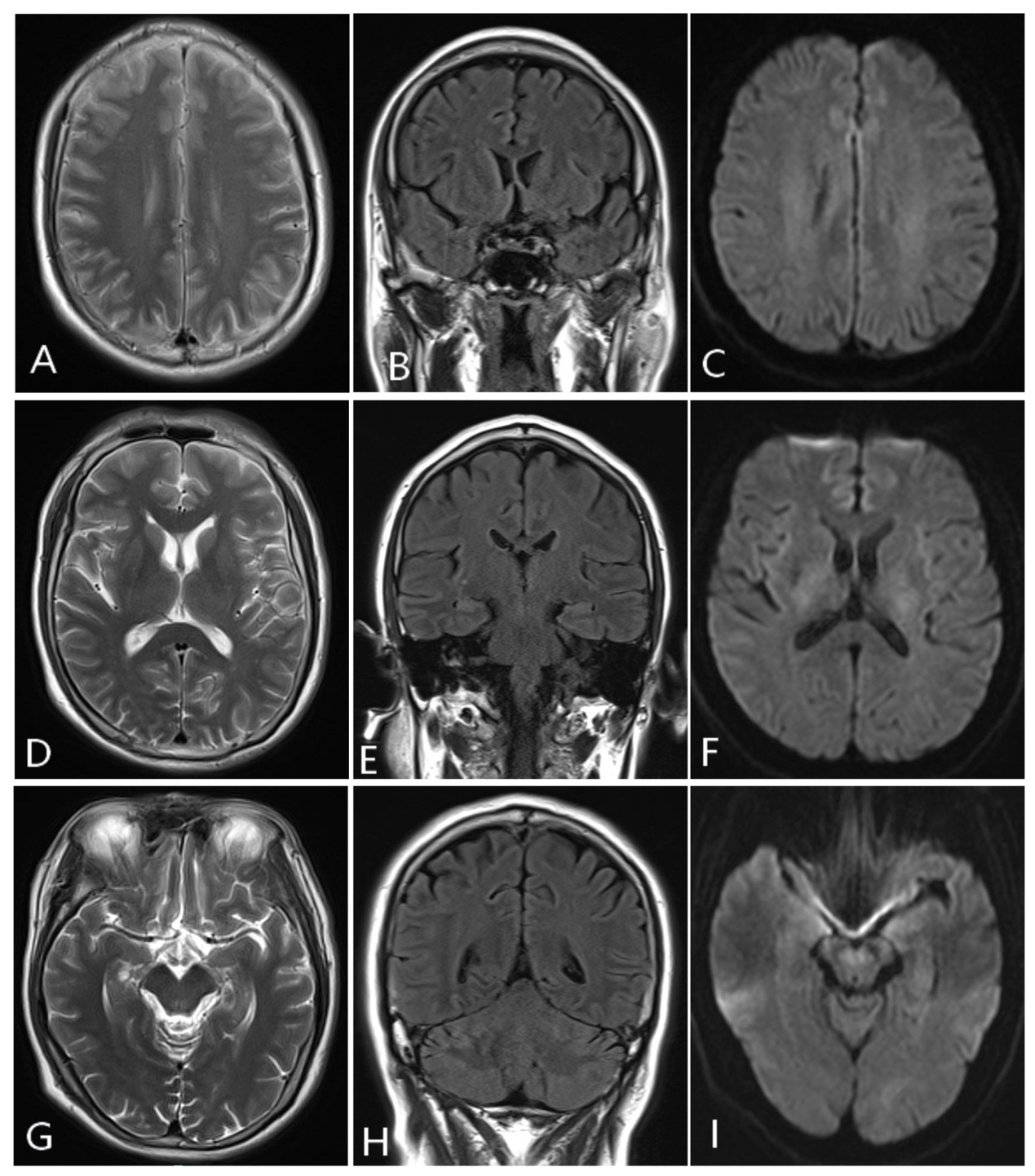


**Figure S9.** The cerebral MRI in patient 9 with positive NMDAR, GABA_B_R and GAD65 antibodies.
